# Baicalein self‐microemulsion based on drug–phospholipid complex for the alleviation of cytokine storm

**DOI:** 10.1002/btm2.10357

**Published:** 2022-06-20

**Authors:** Hengfeng Liao, Jun Ye, Yue Gao, Chunfang Lian, Lu Liu, Xiaoyan Xu, Yu Feng, Yanfang Yang, Yuqi Yang, Qiqi Shen, Lili Gao, Zhihua Liu, Yuling Liu

**Affiliations:** ^1^ State Key Laboratory of Bioactive Substance and Function of Natural Medicines, Institute of Materia Medica Chinese Academy of Medical Sciences and Peking Union Medical College Beijing China; ^2^ Beijing Key Laboratory of Drug Delivery Technology and Novel Formulation, Institute of Materia Medica Chinese Academy of Medical Sciences and Peking Union Medical College Beijing China; ^3^ Research and Development Department Beijing Wehand‐Bio Pharmaceutical Co. Ltd Beijing China

**Keywords:** baicalein, cytokine storm, lymphatic transport, phospholipid complex, SMEDDS

## Abstract

Cytokine storm is a phenomenon whereby the overreaction of the human immune system leads to the release of inflammatory cytokines, which can lead to multiple organ dysfunction syndrome. At present, the existing drugs for the treatment of cytokine storm have limited efficacy and severe adverse effects. Here, we report a lymphatic targeting self‐microemulsifying drug delivery system containing baicalein to effectively inhibit cytokine storm. Baicalein self‐microemulsion with phospholipid complex as an intermediate carrier (BAPC‐SME) prepared in this study could be spontaneously emulsified to form 12‐nm oil‐in‐water nanoemulsion after administration. And then BAPC‐SME underwent uptake by enterocyte through endocytosis mediated by lipid valve and clathrin, and had obvious characteristics of mesenteric lymph node targeting distribution. Oral administration of BAPC‐SME could significantly inhibit the increase in plasma levels of 14 cytokines: TNF‐α, IL‐6, IFN‐γ, MCP‐1, IL‐17A, IL‐27, IL‐1α, GM‐CSF, MIG, IFN‐β, IL‐12, MIP‐3α, IL‐23, and RANTES in mice experiencing systemic cytokine storm. BAPC‐SME could also significantly improve the pathological injury and inflammatory cell infiltration of lung tissue in mice experiencing local cytokine storm. This study does not only provide a new lymphatic targeted drug delivery strategy for the treatment of cytokine storm but also has great practical significance for the clinical development of baicalein self‐microemulsion therapies for cytokine storm.

**Abbreviations**
ALI: acute lung injury
BA: baicalein
BAPC: baicalein‐phospholipid complex
BAPC‐SME: baicalein self‐microemulsion with phospholipid complex as an intermediate carrier
BA‐SME: traditional baicalein self‐microemulsion directly prepared from baicalein
BE: baicalin
COVID‐19: coronavirus disease 2019
Dex: dexamethasone
DiR: 1,1′‐dioctadecyl‐3,3,3′,3′‐tetramethylindotricarbocyanine iodide
ELISA: enzyme‐linked immunosorbent assay
HE: hematoxylin and eosin
HRP: horseradish peroxidase
IFN: interferon
IL: interleukin
IP‐10: IFN‐γ‐inducible protein‐10
JAK: Janus kinase
KC: keratinocyte‐derived chemokine
LPS: lipopolysaccharide
MCP‐1: monocyte chemoattractant protein‐1
MDC: macrophage‐derived chemokine
MEM: minimum essential medium
MERS‐CoV: Middle East respiratory syndrome
MIP‐1: macrophage inflammatory protein‐1
NP: nucleoprotein
PBS: phosphate‐buffered saline
qPCR: quantitative polymerase chain reaction
SMEDDS: self‐microemulsifying drug delivery system
STAT: signal transducer and activator of transcription
TNF: tumor necrosis factor
TRITC: tetramethylrhodamine
TTBS: Tris‐buffered saline with Tween 20
VEGF: vascular endothelial growth factor

## 1 INTRODUCTION

**FIGURE 1:**
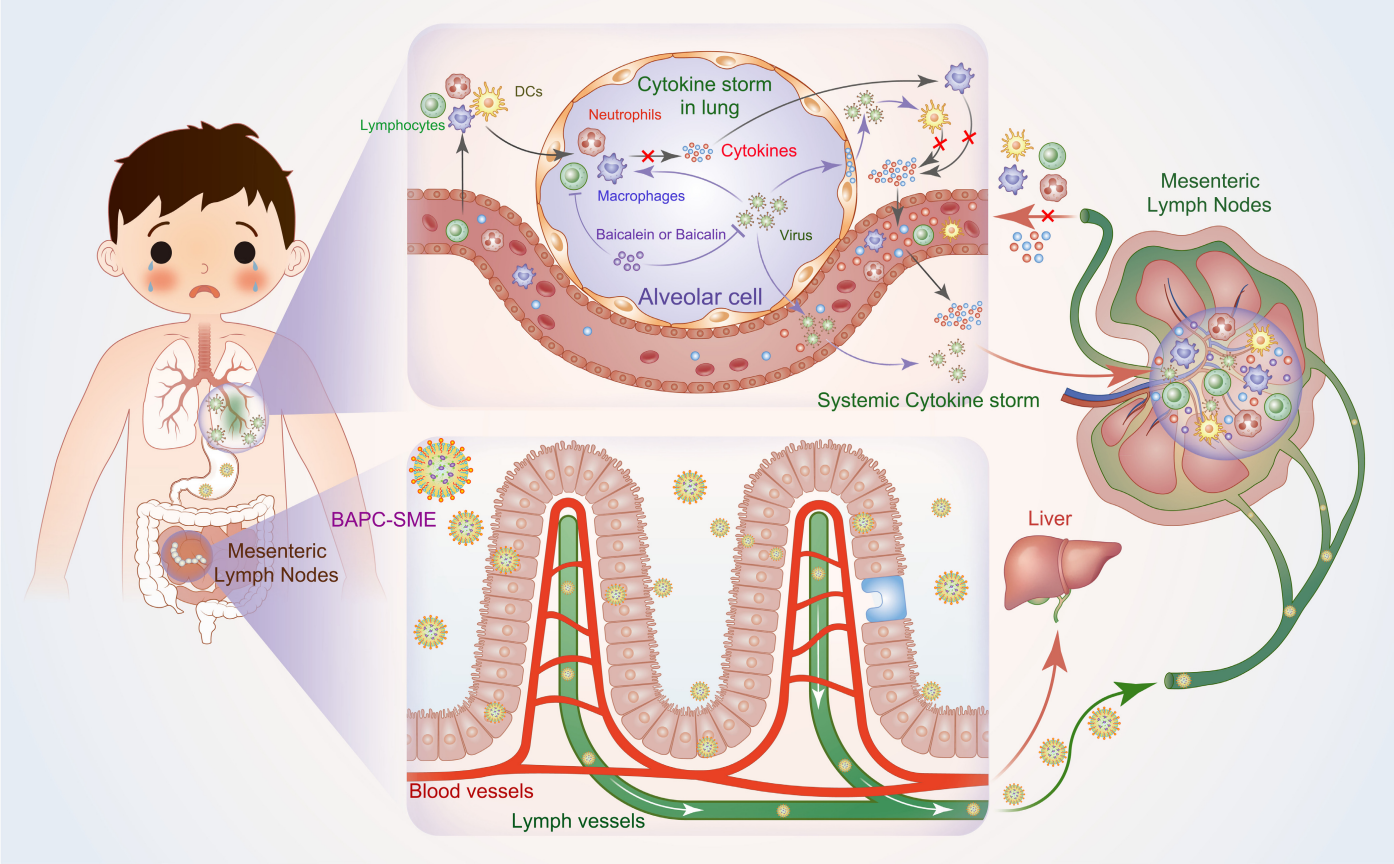
Schematic diagram of the formation of cytokine storm and the effect of BAPC‐SME. When lungs are infected by certain viruses or bacteria, macrophages and dendritic cells in the lung tissue could be over‐activated. Lymphocytes, neutrophils, and other inflammatory cells would be recruited from the blood into the lung. Further, these immune cells secrete a large number of inflammatory cytokines, thus forming local cytokine storm. At the same time, these cytokines and viruses could leak into the systemic circulation and enter the mesenteric lymph nodes (the largest lymph nodes in the body) with the blood. They activate the immune cells in the lymph nodes and promote their proliferation and differentiation, resulting in the secretion of a large number of pro‐inflammatory cytokines. These differentiated immune cells and proinflammatory cytokines are transported into the systemic circulation through the lymphatic system, thus causing systemic cytokine storm. This would cause inflammatory damage to tissues and organs. After oral administration, BAPC‐SME was spontaneously emulsified in the gastrointestinal tract to form nanoscale emulsion droplets, which then targeted the mesenteric lymph nodes through lymphatic transport. Therefore, baicalein could directly act on the immune cells in the lymph nodes and inhibit the excessive expression of inflammatory cytokines. In addition, upon entering the systemic circulation, baicalein and its metabolite baicalin could also inhibit the secretion of cytokines by immune cells in lung tissue, and exhibited an inhibitory effect on viruses in tissues.

The cytokine storm refers to an excessive immune and inflammatory response to external stimuli, characterized by excessive proliferation of T cells and macrophages and the rapid release of inflammatory cytokin. It can be caused by many infectious and non‐infectious diseases, such as influenza, coronavirus disease (COVID‐19), septicemia, and lupus erythematosus, as well as chimeric antigen receptor T cell therapy.^1^, ^2^, ^3^, ^4^, ^5^ The occurrence of cytokine storm can accelerate disease progression, leading to tissue and organ damage, as well as multiple organ dysfunction syndrome.^6^ Some studies have shown that reducing the occurrence of cytokine storm among patients with infectious or non‐infectious diseases can help to reduce organ damage and slow down disease progression, which is particularly important in the treatment of critically ill patients (Figure 1).^7^, ^8^

When cytokine storm result from infection, the levels of proinflammatory cytokines represented by interferon (IFN)‐λ, tumor necrosis factor (TNF)‐α, interleukin (IL)‐1β, IL‐2, IL‐5, IL‐6, IL‐7, IL‐8, granulocyte colony‐stimulating factor (G‐CSF), granulocyte macrophage colony‐stimulating factor (GM‐CSF), vascular endothelial growth factor (VEGF), monocyte chemoattractant protein‐1 (MCP‐1), IFN‐γ‐inducible protein‐10 (IP‐10), and macrophage inflammatory protein‐1 (MIP‐1) were significantly increased in vivo. This is also the main cause of multiple organ dysfunction and failure caused by cytokine storm.^8^, ^9^, ^10^, ^11^, ^12^, ^13^ The lymph nodes and spleen are mainly composed of T and B lymphocytes, contain a large number of macrophages and dendritic cells, and constitute the main sites of occurrence of immune reactions in vivo.^14^, ^15^, ^16^, ^17^, ^18^ When the presence of certain infection, immune cells in the lymph nodes and spleen are over‐activated, proliferate, differentiate, and secrete a large number of proinflammatory cytokines, thereby leading to systemic cytokine storm.

At present, glucocorticoids, cyclooxygenase inhibitors, TNF inhibitors, IL‐1 antagonists, and IL‐6 antagonists are mainly used in the treatment of cytokine storm.^4^, ^7^ However, their curative effects are limited and their adverse effects are severe. For example, glucocorticoids can nonspecifically inhibit cytokine storm; however, their long‐term use can easily cause adverse effects such as double infections, diabetes, osteoporosis, hypertension, and osteonecrosis. Therefore, the development of new safe and effective drugs that regulate cytokine storm is of great significance to save patients' lives.

*Scutellaria baicalensis* has been used as a traditional Chinese medicinal plant for thousands of years and has a significant immunocgqzressive effect.^19^ Baicalein and baicalin are the main bioactive components extracted from the root of *S. baicalensis*.^20^, ^21^, ^22^ At present, a large number of studies have shown that baicalein and its plasma metabolite baicalin do not only have antibacterial and antiviral activities, but also directly act on lymphocytes, macrophages, mast cells, neutrophils, and other immune cells to inhibit the production of biomarkers of the cytokine storm, such as IL‐1β, IL‐6, IFN‐γ, and TNF‐α, thus playing anti‐inflammatory and immunomodulatory roles.^23^, ^24^, ^25^, ^26^, ^27^, ^28^, ^29^, ^30^, ^31^ In addition, clinical studies have shown that high‐dose oral administration of baicalein was safe and well tolerated.^32^ Therefore, we put forward a conjecture: can baicalein and its metabolite baicalin be used in the treatment of cytokine storm caused by infection? However, baicalein itself is insoluble in water and slightly soluble in chloroform, and its oral bioavailability is low. It belongs to Class II under the Biopharmaceutical Classification System. At present, there is no commercially available active monomeric formulation of baicalein. Therefore, it is a great challenge to overcome multiple biological barriers and deliver baicalein to the lymphoid system where immune cells gather to play the function of anti‐inflammatory and immune regulation.

A self‐microemulsifying drug delivery system (SMEDDS), as a lipid carrier composed of an oil phase, emulsifier, and co‐emulsifier, can be diluted and emulsified into an oil‐in‐water nanoemulsion by gastrointestinal fluid after oral administration.^33^ Compared with other new delivery systems, SMEDDS can not only significantly increase the plasma concentration of insoluble drugs, but also significantly promote the lymphatic transport of drugs, and achieve targeted enrichment of drugs in mesenteric lymph nodes.^8^ Therefore, in the past few years, self‐emulsifying drug delivery systems have been widely used to improve the oral bioavailability of insoluble natural drugs and lymphoid absorption of immunomodulatory drugs, such as cyclosporine soft capsules (Novartis) and celecoxib oral solutions (Dr. Reddy's Laboratories).^34^, ^35^, ^36^

In a previous study, we used a baicalein phospholipid complex as an intermediate carrier to construct a baicalein‐phospholipid complex SMEDDS (BAPC‐SME) for the first time.^37^ Our study showed that BAPC‐SME could significantly improve the oral bioavailability of baicalein. The C_max_ of the BAPC‐SME was 7.7 times and 1.9 times higher than those of the baicalein suspension (BA) and conventional baicalein SMEDDS (BA‐SME), respectively. Also, the relative bioavailabilities of BAPC‐SME were 448.7% and 131.0%, relative to BA and BA‐SME, respectively. In addition, we also firstly studied the lymphatic transport of BAPC‐SME. Our results showed that BAPC‐SME could increase the lymphatic transport ratio of baicalein after oral administration from 18.8% for BA and 56.2% for BA‐SME to 70.2%. We suggested that the use of self‐emulsifying nanocarriers for the encapsulation and delivery of baicalein could improve the transmembrane transport ability of drugs by promoting intestinal absorption and lymphatic transport, and promote the target delivery of drugs to mesenteric lymph nodes through the lymphatic transport pathway. Thus, it is beneficial in the use of baicalein to regulate the inflammatory function of immune cells and is of breakthrough significance to enhance the treatment of cytokine storm with oral baicalein. Unfortunately, in our previous study, we did not investigate the effect of BAPC‐SME on cytokine storm in vivo.

The existing studies on SMEDDS have focused on improving the solubility and bioavailability of insoluble drugs; however, there is a lack of in‐depth and systematic research on lymph node targeting by SMEDDS and its improvement of the therapeutic management of cytokine storm.^38^ The application of baicalein self‐microemulsion in the treatment of cytokine storm has not been reported. Thus, based on the results of previous studies, the present study established mouse models of systemic cytokine storm and local lung cytokine storm to investigate the regulation of cytokine storm by BA, BA‐SME, and BAPC‐SME. The changes in the plasma concentrations of inflammatory cytokines among mice with systemic cytokine storm induced by lipopolysaccharide (LPS) were detected and analyzed for the first time. In addition, the present study also investigated the in vitro antibacterial and antiviral activities of BAPC‐SME, the targeting of mesenteric lymph nodes by BAPC‐SME, and explored the mechanism of uptake and transport of BAPC‐SME by Caco‐2 cells.

## 2 MATERIALS AND METHODS

### 2.1 Materials

Baicalein (98% purity) was purchased from Nanjing Zelang Biological Technology Co. Ltd. Soybean phospholipids were purchased from Shanghai Tywei Pharmaceutical Co. Ltd. Ethylis oleas was purchased from Jiangxi Alpha High‐Tech Pharmaceutical Co., Ltd. Tween 80 was cgqzlied by Shanghai Macklin Biochemical Co., Ltd. Transcutol HP was kindly donated by Gattefossé. Ascorbic acid and tetrahydrofuran were cgqzlied by Sinopharm Chemical Reagent Co., Ltd. Minimum Essential Medium (MEM), fetal bovine serum, non‐essential amino‐acid, L‐glutamine, penicillin, streptomycin, and 0.25% trypsin were purchased from Thermo Fisher Scientific. Coumarin 6 (Cou‐6), methyl‐β‐cyclodextrin, chlorpromazine hydrochloride, genistein, and amiloride hydrochloride were purchased from Sigma‐Aldrich. Brefeldin A, monensin, nocodazole, and 4,6‐diamino‐2‐phenyl indole (DAPI) were purchased from Beyotime Biotechnology Co., Ltd. Bafilomycin A1 was cgqzlied by Meilunbio. 1,1′‐Dioctadecyl‐3,3,3′,3′‐tetramethylindotricarbocyanine iodide (DiR) was obtained from AAT Bioquest Inc. Tetramethylrhodaminyl (TRITC) phalloidin was purchased from Yeasen Biotechnology Co., Ltd.

### 2.2 Cell culture and animals

The Caco‐2 cell line was cgqzlied by the Cell Resource Center, Peking Union Medical College (Beijing, China). Caco‐2 cells were cultured in MEM supplemented with 10% (vol/vol) fetal bovine serum, 1% (vol/vol) non‐essential amino‐acid, 1% (vol/vol) L‐glutamine, 100 U/ml penicillin, and 100 μg/ml streptomycin. All cells were maintained at 37°C in a humidified chamber with 5% CO_2_.

All animal protocols were approved by the Institutional Animal Care and Use Committee of Peking Union Medical College. The care of laboratory animals and animal experimental operations were performed in accordance with the Administration Regulations on Laboratory Animals of Beijing Municipality. Male Sprague–Dawley rats (weight, 250 ± 20 g) and C57BL/6 mice (weight, 20 ± 2 g) were purchased from Beijing Huafukang Bioscience Co. Inc. Seven days prior to the experiment, the animals were maintained in standard air‐conditioned cages with alternating 12 h light/dark cycles at a room temperature of 25 ± 2°C, and fed with standard animal chow with free access to water. The animals were allowed to fast for 12 h, with free access to water before the experiment.

### 2.3 Preparation and characterization of baicalein self‐microemulsion based on phospholipid complexes

As described in a previous study,^37^ BAPCs were prepared with BA and phospholipids in a mass ratio of 1:3.5 (wt/wt) using the solvent evaporation method. To prepare BAPC‐SME, ethyl oleate, Tween 80, and Transcutol HP were weighed and mixed in a mass ratio of 2:5:3, after which BAPC was added. The mixture was shaken in an air bath oscillator overnight to obtain a transparent solution. BAPC‐SME loaded with Cou‐6 or DiR was obtained by dissolving fluorescent dye into the BAPC‐SME.

According to the method of characterization of self‐microemulsions described in a previous study,^37^ the content, particle size, and zeta potential of the prepared BAPC‐SME were simply characterized.

### 2.4 Distribution of BAPC‐SME in mesenteric lymph nodes

All rats were randomly divided into two groups and given DiR solution and DiR‐labeled BAPC‐SME at a dose of 1 mg/kg. The rats were sacrificed 0.5 and 2.0 h after administration, and their small intestines and mesenteric lymph nodes were excised. After washing with normal saline, the samples were subjected to fluorescence imaging in vitro using an IVIS Spectrum In Vivo Small Animal Imager (PerkinElmer).

Eighteen male Institute of Cancer Research mice were randomly divided into two groups. One group was given Cou‐6 solution and the other was given Cou‐6‐labeled BAPC‐SME intragastrically at a dose of 1 mg/kg. After 0.5, 1, and 2 h, the mice were sacrificed. Their mesenteric lymph nodes were excised and prepared into a single cell suspension using a cell sieve, after which their fluorescence intensity was detected using a C6 Flow Cytometer (BD Biosciences).

### 2.5 Uptake of BAPC‐SME by Caco‐2 cells

Caco‐2 cells seeded in 12‐well plates were cultured at 37°C for 24 h, after which the cells were incubated with the solutions (100 ng/ml) of Cou‐6 and Cou‐6‐labeled BAPC‐SME in a constant temperature shaker at 37°C for 0.5, 1.0, and 1.5 h, respectively. Then, the cells were washed with phosphate‐buffered saline (PBS) and digested with 0.25% trypsin. Finally, the cell suspension was analyzed using a C6 Flow Cytometer (BD Biosciences). In addition, Caco‐2 cells seeded in a confocal dish were incubated with the solutions (100 ng/ml) of Cou‐6 and Cou‐6‐labeled BAPC‐SME in a shaker at a constant temperature of 37°C for 1 h. Another group of Caco‐2 cells was pretreated at 4°C for 1 h, and then incubated with a pre‐cooled Cou‐6‐labeled BAPC‐SME solution at 4°C for 1 h. After incubation, the cells were washed with PBS, fixed with 4% paraformaldehyde and 0.5% Triton X‐100, stained with TRITC phalloidin and DAPI, and finally observed using an FV1000 laser confocal microscope (Olympus).

### 2.6 Cellular uptake and intracellular transport pathways of BAPC‐SME in Caco‐2 cells

To investigate the cellular uptake and intracellular transport pathways of BAPC‐SME in Caco‐2 cells, the cells were pretreated with methyl‐β‐cyclodextrin, chlorpromazine hydrochloride, genistein, amiloride hydrochloride, brefeldin A, monensin, nocodazole, and bafilomycin A1 at 37°C for 1 h. Then, Cou6‐labeled BAPC‐SME solutions with different inhibitors at the same concentrations as described were added and incubated at 37°C for another 1 h. After incubation, all cells were treated and analyzed as previously described via flow cytometry and laser confocal microscopy.

### 2.7 Lipopolysaccharide‐induced systemic cytokine storm model

Male C57 mice were randomly divided into seven groups and fasted for 12 h before the experiment. The mice in the model group underwent intraperitoneal injection with LPS (5 mg/kg) to induce a cytokine storm, while the mice in the blank control group were injected with the same volume of normal saline. The eyeballs of the mice were removed and blood samples were collected at 1.5, 4, 6, 8, 12, and 24 h after the administration of LPS. Blood samples were placed in tubes containing heparin sodium. Then, the blood samples were centrifuged for 10 min at 1000*g*. The supernatant was extracted and stored at −80°C. The concentrations of IL‐1α, IFN‐γ, TNF‐α, MCP‐1, IL‐12p70, IL‐10, IL‐27, IL‐23, IL‐17A, and IFN‐β in the plasma were measured using the Mouse Inflammation Panel (Cat. no. 740446, BioLegend). And MIG, MIP‐1α, MIP‐1β, and IL‐4 were measured using the Mouse Cytokine Release Syndrome Panel (Cat. no.741024, BioLegend). IL‐6 was measured using the ELISA (Cat. no. E‐EL‐M0021c, Elabscience).

### 2.8 Inhibitory effect on systemic cytokine storm

To determine the effective dose of BAPC‐SME, male C57 mice were randomly divided into seven groups and fasted for 12 h before the experiment. Both the blank control and model groups were given normal saline orally, while the experimental groups were given orally different doses of BAPC‐SME diluted with water (20, 40, 80, and 160 mg/kg). And the positive drug group was injected intraperitoneally dexamethasone dissolved in normal saline (30 mg/kg). After 40 min, both the model and experimental groups underwent intraperitoneal injection with LPS (5 mg/kg) to induce systemic cytokine storm, while the blank control group was injected with the same volume of normal saline. After 4 h, the eyeballs of the mice were excised and blood samples were collected. Then, the blood samples containing heparin were centrifuged for 10 min at 1000*g*. The supernatant was extracted and stored at −80°C. The concentration of IL‐6 in plasma was determined via ELISA (Cat. no. E‐EL‐M0021c, Elabscience).

Further, the inhibitory effects of different formulations on cytokine storm were investigated. Male C57 mice were randomly divided into six groups: the control, model, BA, BA‐SME, BAPC‐SME, and dexamethasone (positive drug) groups. First, they were orally given normal saline, BA (dispersed in 0.1% CMC‐Na, 80 mg/kg), BA‐SME (diluted with water, 80 mg/kg), and BAPC‐SME (diluted with water, 80 mg/kg), respectively. And the mice in positive drug group were injected intraperitoneally dexamethasone (30 mg/kg). Then, LPS (5 mg/kg) was injected intraperitoneally to induce systemic cytokine storm after 40 min, while the same volume of normal saline was injected in the control group. The eyeballs of the mice were excised and blood samples were collected at 1.5 h (IL‐6 and TNF‐α), 4 h (IL‐1β, IL‐10, IL‐12, IL‐23, IFN‐β, MIP‐3α, MIG, RANTES, MIP‐1β, MIP‐1α, Eotaxin, keratinocyte‐derived chemokine [KC], TARC, BLC, VEGF, LIX, and macrophage‐derived chemokine [MDC]), and 6 h (IL‐1α, IL‐17A, IL‐27, MCP‐1, IFN‐γ, and GM‐CSF) after LPS injection. The blood samples containing heparin were centrifuged at 1000*g* at 2–8°C for 10 min and the supernatant was stored at −20°C. The mice were sacrificed at 1.5 h after LPS injection, and the mesenteric lymphoid nodes were excised and stored at −80°C. The concentrations of IL‐1α, IL‐1β, IL‐10, IL‐12, IL‐17A, IL‐23, IL‐27, IFN‐γ, IFN‐β, MCP‐1, and GM‐CSF in the plasma were measured using the Mouse Inflammation Panel (Cat. no. 740446, BioLegend). And MIG, MIP‐1β, and VEGF were measured using the Mouse Cytokine Release Syndrome Panel (Cat. no.741024, BioLegend). RANRES, MIP‐3α, MIP‐1α, Eotaxin, KC, TARC, BLC, LIX, and MDC were measured using the Mouse Proinflammatory Chemokine Panel (Cat. no. 740451, BioLegend). IL‐6 and TNF‐α were measured using the ELISA (Cat. no. E‐EL‐M0044c and 49c, Elabscience).

### 2.9 Expression of inflammatory cytokines in mesenteric lymph nodes

The mesenteric lymph nodes were homogenized with Trizol, after which total intracellular RNA was extracted using an RNeasy Mini Kit. Reverse transcriptional amplification was performed using an EvoM‐MLV reverse transcription kit. The cDNA products obtained by reverse transcription were diluted and used as quantitative polymerase chain reaction (qPCR) templates to detect the expressions of the mRNA of TNF‐α, IL‐1β, IL‐6, IL‐12B, MCP‐1, and TGF‐β in mesenteric lymph nodes via quantitative real‐time PCR assay.

### 2.10 Western blot analysis

The mesenteric lymph nodes were mixed with radioimmunoprecipitation assay buffer containing protease and phosphatase inhibitors, and ground. The supernatant was extracted as a protein sample after centrifugation (12,000 rpm) at 4°C. The protein samples were separated using a Mini‐300 electrophoresis device (Major Science) using sodium dodecyl‐sulfate polyacrylamide gel electrophoresis gel, and electro‐blotted onto nitrocellulose membranes. The membranes were blocked with Tris‐buffered saline with Tween 20 (TTBS) blocking solution containing 5% skimmed milk powder. Then, the membranes were incubated with primary antibodies overnight at 4°C. The primary antibody probes used in this experiment were NF‐κB p65 monoclonal antibody (1:3000), NF‐κB p65 (phospho Ser536) antibody (1:2000), Stat3 polyclonal antibody (1:2000), Stat3 (phospho Tyr705) polyclonal antibody (1:2000), IκBα (phospho‐Ser32) rabbit polyclonal antibody (1:2000), JAK2 (phospho Tyr1007) polyclonal antibody (1:1000), and β‐actin monoclonal antibody (1:8000). After incubating the primary antibodies, the membranes were rinsed with TTBS, and then incubated with secondary antibody at room temperature for 1 h. The secondary antibody probes in this experiment were Fredo Polyclonal Rabbit Anti‐Mouse IgG(H+L) Antibody Horseradish Peroxidase (HRP) Conjugate (1:14,000) and Fredo Polyclonal Goat Anti‐Rabbit IgG(H+L) Antibody HRP Conjugate (1:14,000). After incubating the secondary antibody, the membranes were developed using an enhanced chemiluminescence substrate and ChemiDoc Fluorescence XRS (Bio‐Rad Laboratories) + chemiluminescence image analyzer (Bole Life Medical Products Co., Ltd.).

### 2.11 Inhibitory effect on local cytokine storm in the lung

Male C57 mice were randomly divided into seven groups: the control, model, BA, BA‐SME, BAPC‐SME, and dexamethasone (positive drug) groups. First, they were given normal saline, different baicalein formulations (80 mg/kg), and dexamethasone (30 mg/kg). Then, LPS (5 mg/kg) was injected intratracheally to induce local cytokine storm in the lung after 40 min. The mice were sacrificed at 6 h after LPS injection, and the lung tissue was excised for hematoxylin and eosin (HE) staining, immunohistochemistry, and qPCR assay.

### 2.12 Cytokine levels in lung tissue

Lung tissue was homogenized with Trizol, and total intracellular RNA was extracted using an RNeasy Mini Kit. Reverse transcriptional amplification was performed using an EvoM‐MLV reverse transcription kit. The cDNA products obtained by reverse transcription were diluted and used as qPCR templates to detect the expression levels of the mRNA of TNF‐α, MCP‐1, IL‐1β, IL‐6, IL‐10, and IFN‐γ in lung tissue via quantitative real‐time PCR assay.

### 2.13 Histopathology

The lung tissue samples were fixed with 4% paraformaldehyde, embedded in paraffin, and sliced. After that, the samples were stained with hematoxylin solution (HE staining). Finally, pathological examination was performed under a microscope. Destruction of alveolar structures, edema of the alveolar septum, thickening of the alveolar wall, infiltration by inflammatory cells, telangiectasia and hyperemia, destruction of the bronchi, and mucus secretion in the cavity were observed.

### 2.14 Immunohistochemistry

The lung tissue samples were fixed with 4% paraformaldehyde, dehydrated, embedded in paraffin, and sliced. After antigen repair and blocking of endogenous peroxidase, lung tissue sections were blocked in blocking solution for 1 h. The sections were incubated with anti‐CD3 antibody (1:200) or anti‐CD68 antibody (1:200) overnight at 4°C to detect tissue T cells and macrophages, and then incubated with biotinylated sheep anti‐rabbit IgG antibody. Finally, the sections were incubated with 3,3*′*‐diaminobenzidine chromogenic solution, followed by re‐staining with hematoxylin. The stained tissue sections were observed under a microscope and the number of brown granulosa cells in different groups were compared.

### 2.15 Statistical analysis

All results are expressed as the mean ± standard error of the mean. The statistical analyses were carried out using independent‐samples *t* tests for two groups with SPSS version 17.0 (IBM Corporation), and one‐way analysis of variance for multiple groups with GraphPad Prism V.7.00 for Windows (GraphPad Software). Differences were considered to be statistically significant at *p* < 0.05.

## 3 RESULTS

### 3.1 Characterization of BAPC‐SME

**FIGURE 2:**
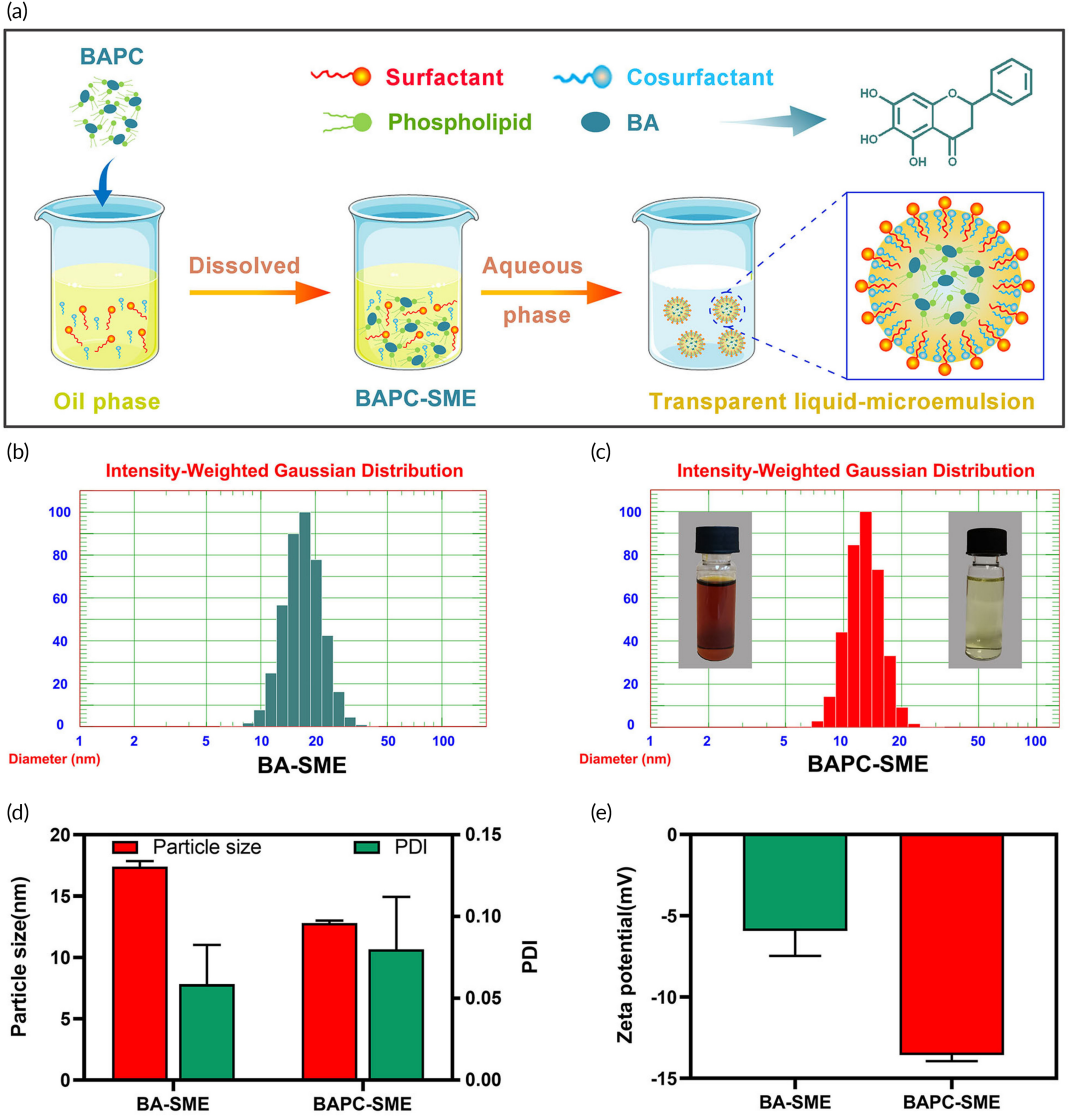
Characterization of BA‐SME and BAPC‐SME. (a) Schematic diagram of the preparation of BAPC‐SME. (b–e) Droplet size distribution, average droplet size, polydispersion index, and zeta potential of BA‐SME and BAPC‐SME. (c) Concentrate (left) and microemulsion (right) appearance of BAPC‐SME

The results showed that the mean concentration of baicalein in BAPC‐SME was 21.04 ± 0.25 mg/g. When BAPC‐SME was mixed with water, the concentrate was completely emulsified in 1 min. The average droplet size was 12.8 ± 0.2 nm with a polydispersity index of 0.08 ± 0.03, and zeta potential of −13.57 ± 0.38 mV (Figure 2). Compared with the results of a previous study,^37^ the particle size and zeta potential of BAPC‐SME changed slightly, which may be related to the differences in suppliers of ethyl oleate and Tween 80.

### 3.2 Distribution of BAPC‐SME in mesenteric lymph nodes

**FIGURE 3:**
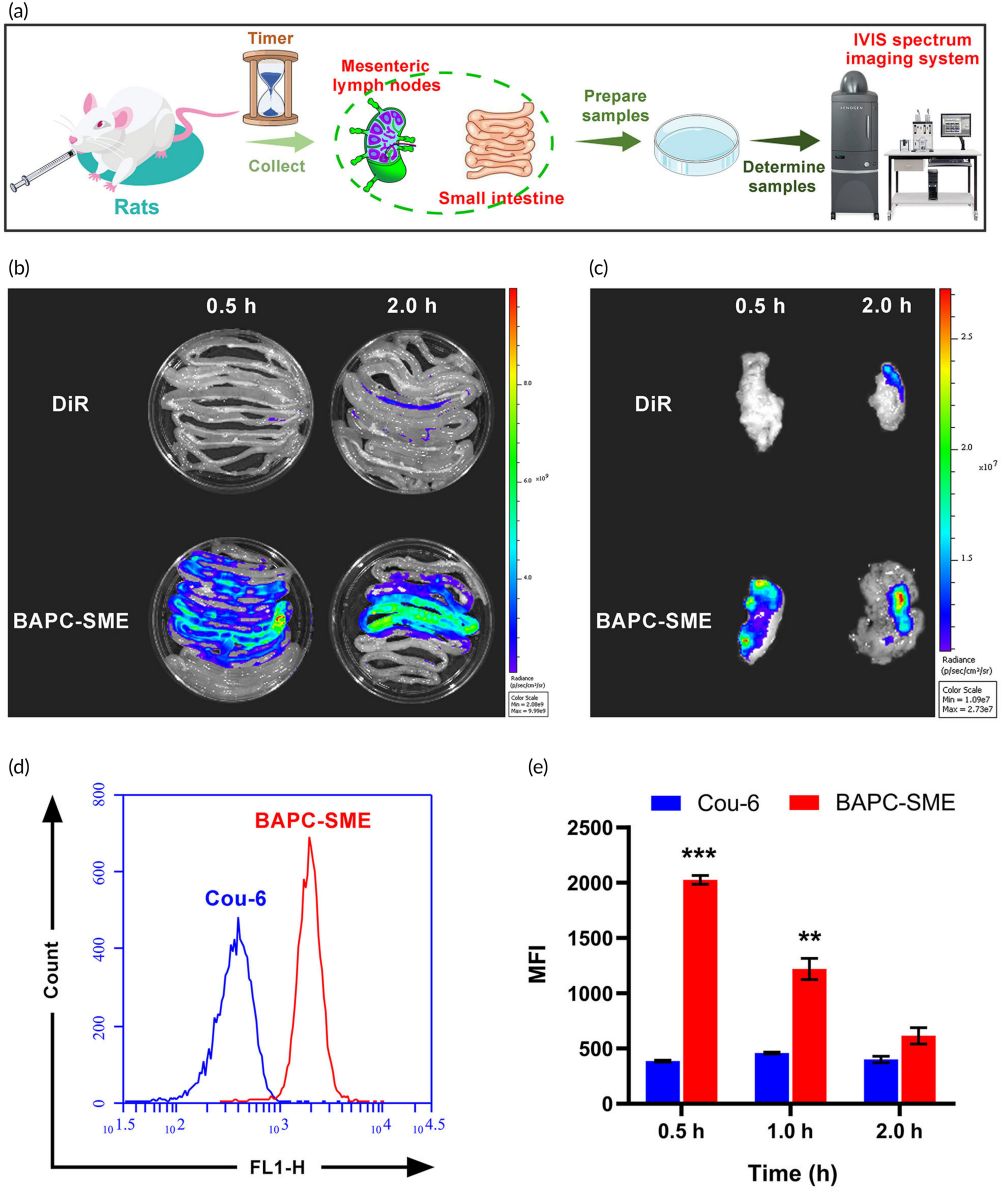
Biodistribution of BAPC‐SME. (a) The schematic of treatment. (b,c) In vitro fluorescence imaging of DiR‐labeled BAPC‐SME in intestine and mesenteric lymph nodes. (d,e) Distribution of Cou‐6‐labeled BAPC‐SME in mesenteric lymph nodes determined via flow cytometry (*n* = 3). **p < 0.01, ***p < 0.001 compared with Cou‐6

In vitro fluorescence images of intestinal tracts and mesenteric lymph nodes of rats are shown in Figure 3b,c, respectively. The fluorescence intensity of mesenteric lymph nodes and intestinal tissues increased significantly after oral administration of DiR‐labeled BAPC‐SME for 0.5 h, which was significantly higher than that in the DiR solution group. The results suggested that BAPC‐SME could promote the intestinal absorption of drugs and has a significant targeted aggregation in mesenteric lymph nodes.

We also quantitatively studied the distribution of Cou‐6‐labeled BAPC‐SME in mesenteric lymph nodes by flow cytometry (Figure 3d,e). The fluorescence intensity of the mesenteric lymph nodes of mice did not change significantly at 0.5, 1.0, and 2.0 h after oral administration of Cou‐6 solution, and remained at a low level. On the contrary, the fluorescence intensity of mesenteric lymph nodes was significantly increased after oral administration of Cou‐6‐labeled BAPC‐SME, and the fluorescence intensity at 0.5 h was about five times higher than that of the Cou‐6 solution group. However, the concentration of Cou‐6‐labeled BAPC‐SME in mesenteric lymph nodes decreased with time. The results further proved that SMEDDS could significantly increase the distribution of active pharmaceutical ingredients in mesenteric lymph nodes.

### 3.3 Cellular uptake of BAPC‐SME

**FIGURE 4:**
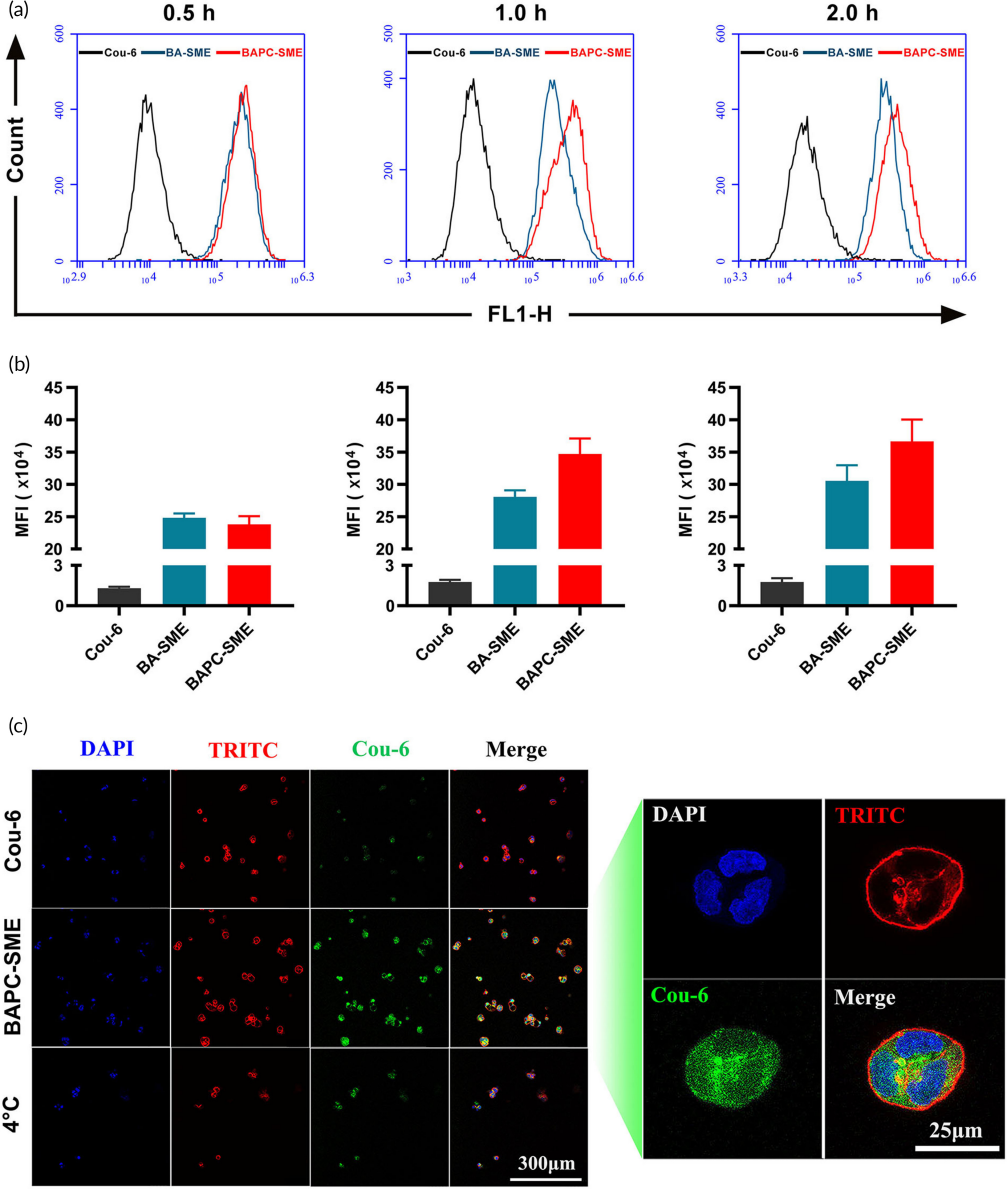
The cellular uptake of BAPC‐SME in Caco‐2 cells. (a,b) Quantification of the cellular uptake of Cou‐6‐labeled BAPC‐SME and BA‐SME at different times via flow cytometry (*n* = 3). (c) Confocal micrographs of the cellular uptake of Cou‐6‐labeled BAPC‐SME in Caco‐2 cells at 37°C and 4°C. The cellular uptake of Cou‐6 solution at 37°C was set as the control.

In this study, Cou‐6‐labeled BAPC‐SME was used for the quantitative analysis of cellular uptake. Flow cytometry was used to detect the fluorescence intensity of Caco‐2 cells to characterize the cellular uptake of BAPC‐SME. Cou‐6‐labeled BAPC‐SME could be obviously absorbed by Caco‐2 cells after incubation for 0.5, 1.0, and 1.5 h (Figure 4a,b) and the cellular uptake of Cou‐6‐labeled BAPC‐SME was significantly higher than that of Cou‐6 solution (*p* < 0.01). Simultaneously, the cellular uptake of Cou‐6‐labeled BAPC‐SME increased gradually and reached saturation with increasing time, but did not increase after 1.0 h.

Further, DAPI and TRITC phalloidin were used to label the nucleus and cytoskeleton, respectively. A laser confocal microscope was used to observe the uptake of Cou‐6‐labeled BAPC‐SME in Caco‐2 cells at 37°C and 4°C. The cellular uptake of Cou‐6‐labeled BAPC‐SME was significantly higher than that of Cou‐6 solution at 37°C (Figure 4c). However, the cellular uptake of Cou‐6‐labeled BAPC‐SME decreased significantly at 4°C. It is known that low temperatures lead to a decrease in the activity of various cellular enzymes, thus inhibiting the transmembrane transport of some substances that consume metabolic energy (adenosine triphosphate). Therefore, we suspected that the cellular uptake of BAPC‐SME depended on energy, and the transmembrane pathway might involve endocytosis.

### 3.4 Cellular uptake and intracellular transport pathways of BAPC‐SME

**FIGURE 5:**
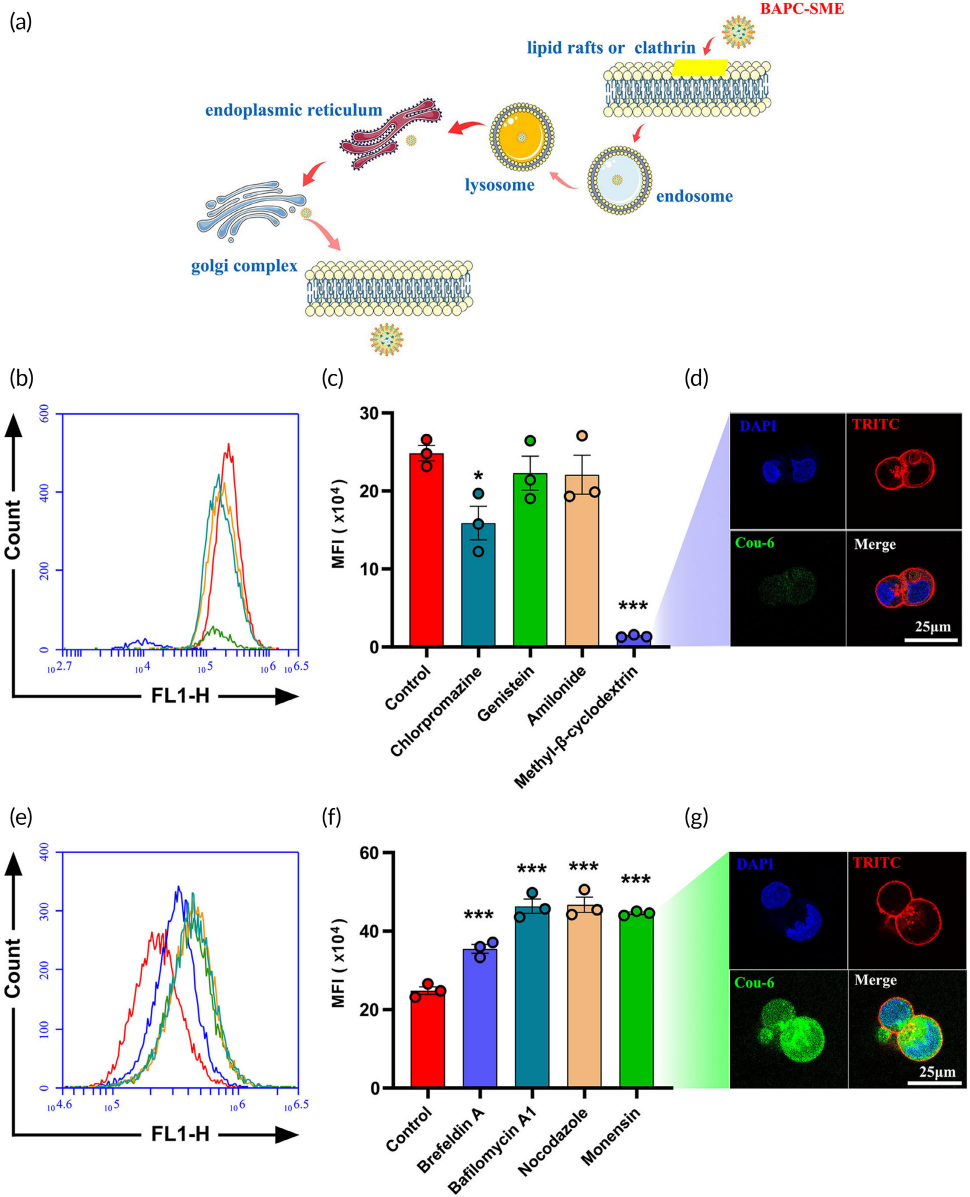
The cellular uptake and intracellular transport pathways of BAPC‐SME in Caco‐2 cells. (a) Schematic diagram of cellular uptake and transport of BAPC‐SME. The influence of different endocytosis inhibitors (b,c) and intracellular transport inhibitors (e,f) on the cellular uptake of Cou‐6‐labeled BAPC‐SME was investigated via flow cytometry. All data were compared to those of Cou‐6‐labeled BAPC‐SME without inhibitors (*p < 0.05, **p < 0.01, ***p < 0.001). The effects of methyl‐β‐cyclodextrin (d) and monensin (g) on the cellular uptake of Cou‐6‐labeled BAPC‐SME were also observed via confocal laser scanning microscopy.

Previous studies have reported that methyl‐β‐cyclodextrin, chlorpromazine, genistein, and amiloride could inhibit cellular endocytosis mediated by lipid raft, clathrin, caveolin, and macropinocytosis, respectively.^39^, ^40^, ^41^, ^42^, ^43^, ^44^ After Caco‐2 cells were incubated with Cou‐6‐labeled BAPC‐SME solution containing methyl‐β‐cyclodextrin, chlorpromazine, genistein, and amiloride for 1 h, methyl‐β‐cyclodextrin and chlorpromazine could significantly inhibit the cellular uptake of Cou‐6‐labeled BAPC‐SME at inhibition rates of 95.2% and 38.7%, respectively (Figure 5b–d). However, the inhibitory effects of genistein and amiloride were not obvious. These results suggested that the cellular uptake pathways of BAPC‐SME by Caco‐2 cells might be mainly related to lipid raft‐ and clathrin‐mediated endocytosis.

In addition, after ingestion by Caco‐2 cells, the microemulsion can be secreted into the tissue space through a series of intracellular transport pathways, thus entering the mesenteric lymphatic system. However, when this process is inhibited, the microemulsion will remain in the cells. Some studies have shown that brefeldin A could block the transport pathway from the endoplasmic reticulum to the Golgi apparatus by triggering the retrograde transport of Golgi enzymes. Monensin inhibits the transport of macromolecules from the Golgi apparatus to the plasma membrane mainly by destroying the Golgi complex.^45^ Nocodazole could bind to β‐tubulin in microtubules to inhibit the formation of disulfide bonds and the dynamic changes in microtubules. It also destroys the structure of the Golgi apparatus and induces Golgi transport disorders.^46^ Bafilomycin A1 can inhibit the maturation of early inclusions to lysosomes, thus interfering with the lysosome‐mediated intracellular transport pathway of nanocarriers.^47^ After Caco‐2 cells were incubated with Cou‐6‐labeled BAPC‐SME solution containing brefeldin A, monensin, nocodazole, and bafilomycin A1 for 1 h, the four inhibitors could significantly increase the concentration of Cou‐6‐labeled BAPC‐SME in Caco‐2 cells, among which monensin had the most obvious effect (Figure 5e–g). Compared with the control group without inhibitors, brefeldin A, monensin, nocodazole, and bafilomycin A1 increased the cellular uptake of Cou‐6‐labeled BAPC‐SME by 45.7%, 178.2%, 88.3%, and 86.6%, respectively. The results suggested that the intracellular transport pathways of BAPC‐SME in Caco‐2 cells mainly included the endosomes, lysosomes, endoplasmic reticula, and Golgi apparatus.

### 3.5 LPS‐induced systemic cytokine storm model

**FIGURE 6:**
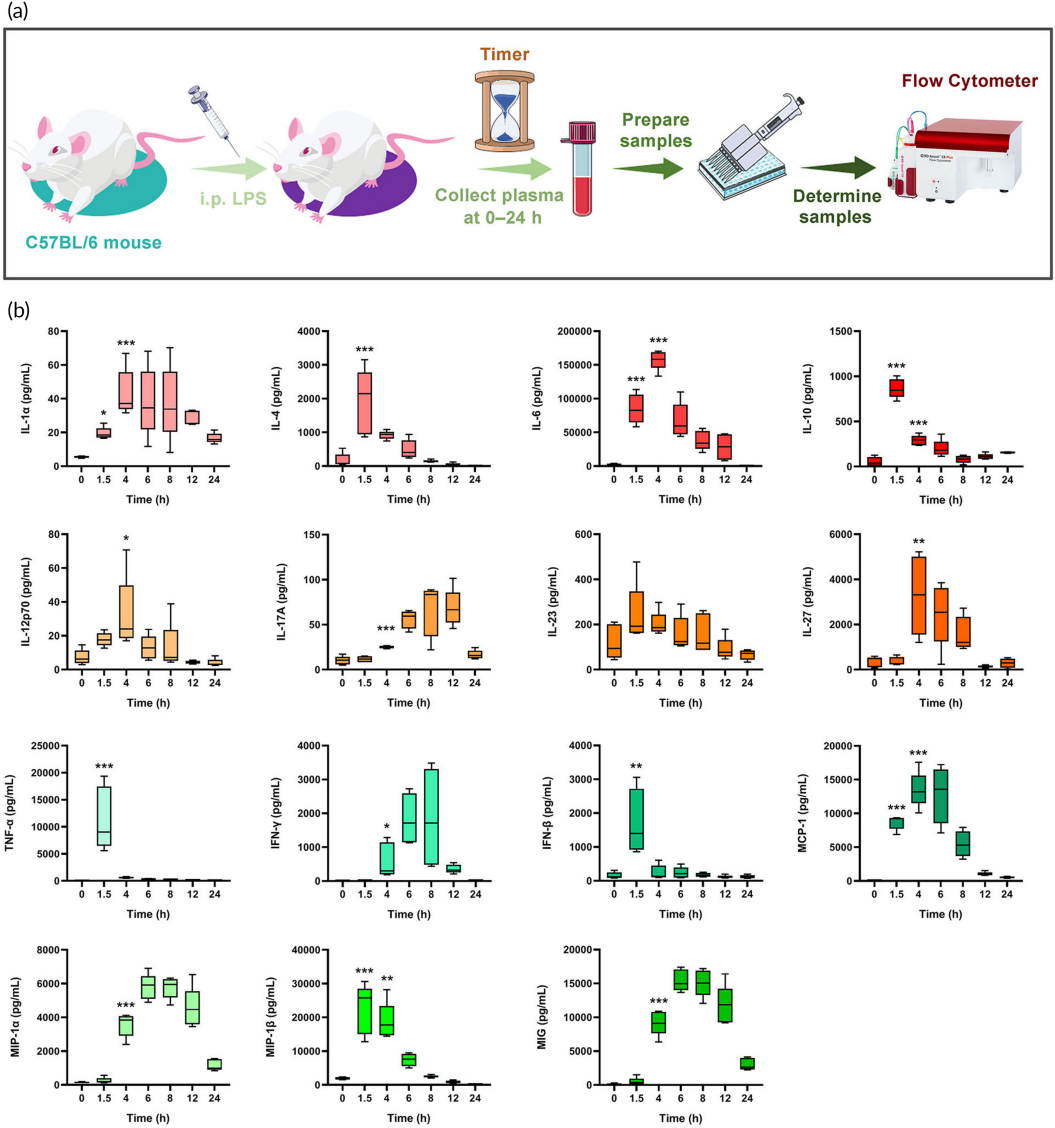
The establishment of systemic cytokine storm model. (a) The schematic of treatment schedule. (b) Mean plasma concentration of TNF‐α, MCP‐1, IL‐27, IL‐17A, IL‐6, IL‐12p70, IL‐10, IL‐1α, MIG, IFN‐β, IFN‐γ, IL‐23, IL‐4, MIP‐1α, and MIP‐1β in C57BL/6 mice at different time after intraperitoneal administration of lipopolysaccharide (5 mg/kg). *n* = 4–5, *p < 0.05, **p < 0.01, ***p < 0.001 compared with 0 h (Table S4).

The concentrations of IL‐1α, IFN‐γ, TNF‐α, MCP‐1, IL‐12p70, IL‐10, IL‐6, IL‐27, IL‐17A, IFN‐β, MIG, MIP‐1α, MIP‐1β, and IL‐4 in the plasma of mice increased significantly after intraperitoneal injection of LPS (P < 0.05) (Figure 6). TNF‐α, IL‐10, IFN‐β, IL‐4, and MIP‐1β reached peak concentrations at 1.5 h after injection of LPS. MCP‐1, IL‐27, IL‐6, IL‐12p70, and IL‐1α reached peak concentrations at 4 h. MIG, IFN‐γ, and MIP‐1α reached peak concentrations at 6 h, while IL‐17A reached its peak concentration at 12 h. The results showed that intraperitoneal injection of LPS could cause significant systemic cytokine storm in mice, suggesting that the model was established successfully. Simultaneously, the plasma cytokines were metabolized gradually with the extension of time, and the plasma concentrations of most cytokines returned to normal levels after 24 h. In addition, the plasma concentration of the inhibitory inflammatory cytokine IL‐10 increased rapidly after the administration of LPS, and maintained a high concentration even after 24 h, which may be one of the reasons why the plasma level of inflammatory cytokine decreased gradually with time.

On one hand, this study confirmed the feasibility of using LPS to establish mouse cytokine storm model for the first time. On the other hand, this experiment also determined the appropriate blood collection time for follow‐up research, in order to more effectively compare the inhibitory effect of different formulations on cytokine storm.

### 3.6 Inhibitory effect on systemic cytokine storm

**FIGURE 7:**
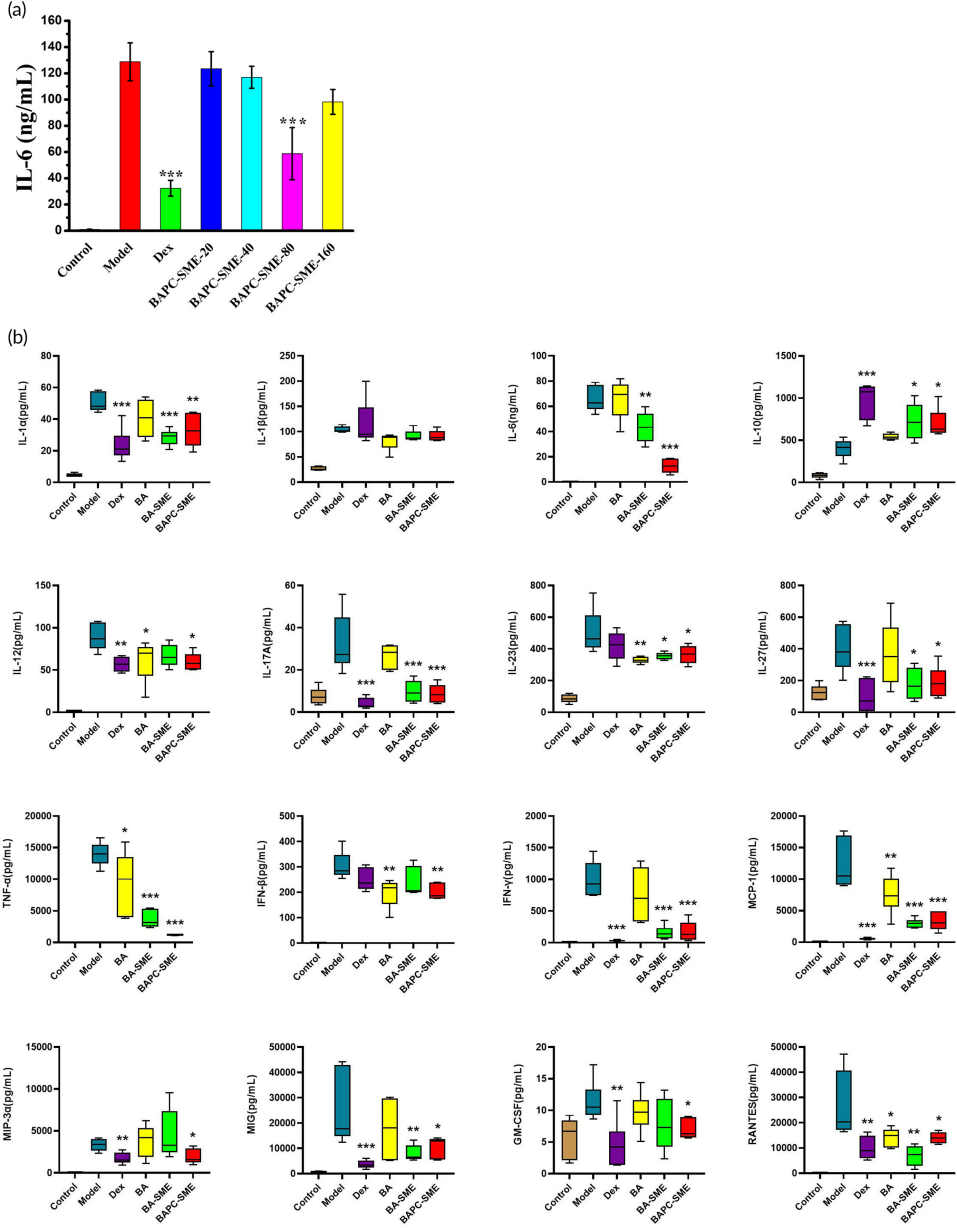
Inhibitory effect of BAPC‐SME on systemic cytokine storm. (a) Mean plasma concentration of IL‐6 in C57BL/6 mice after oral administration of different doses of BAPC‐SME following intraperitoneal injection of lipopolysaccharide (5 mg/kg, Table S5). (b) Mean plasma concentration of cytokine in C57BL/6 mice after oral administration of BA, BA‐SME, and BAPC‐SME following the intraperitoneal injection of lipopolysaccharide (Table S6). *n* = 4–6, *p < 0.05, **p < 0.01, ***p < 0.01 compared with model. The data of other cytokines are not shown in the picture.

The concentration of IL‐6 in the plasma of mice increased by 122.7 times after intraperitoneal injection of LPS, and dexamethasone and 80 mg/kg of BAPC‐SME could significantly inhibit the increase in the concentration of IL‐6 (Figure 7a). However, BAPC‐SME showed no significant effect at doses of 20, 40, and 160 mg/kg. We speculated that the dose of 160 mg/kg was too high, so that BAPC‐SME could not be completely emulsified, thus affecting the absorption of baicalein. Therefore, we chose 80 mg/kg as the effective dose of BAPC‐SME and other baicalein formulations in vivo.

In this study, 18 characteristic cytokines, such as TNF‐α, IL‐1, IL‐6, IFN‐γ, and MCP‐1, were used to investigate the inhibitory effects of different baicalein formulations on systemic cytokine storm. In addition, 7 non‐characteristic inflammatory factors such as MDC and KC were also studied. After intraperitoneal injection of LPS (5 mg/kg), the concentrations of TNF‐α, IL‐1α, IL‐6, IFN‐γ, MCP‐1, IL‐17A, IL‐27, GM‐CSF, MIG, IFN‐β, IL‐12p70, IL‐1β, IL‐10, MIP‐3α, IL‐23, MDC, MIP‐1β, MIP‐1α, Eotaxin, KC, RANTES, TARC, and BLC in plasma were significantly increased; only VEGF and LIX showed no significant changes compared with the blank control group. This suggested that the model was established successfully. Oral administration of BAPC‐SME could significantly inhibit the secretion of 14 inflammatory cytokines, including TNF‐α, IL‐6, IFN‐γ, MCP‐1, IL‐17A, IL‐27, IL‐1α, GM‐CSF, MIG, IFN‐β, IL‐12, MIP‐3α, IL‐23, and RANTES in the plasma of mice in the model (Figure 7b). Their inhibition rates were 91.6%, 70.4%, 74.0%, 58.0%, 64.1%, 32.9%, 34.7%, 38.7%, 61.4%, 33.0%, 33.3%, 41.0%, 27.2%, and 37.7%, respectively. The concentration of the anti‐inflammatory cytokine IL‐10 in plasma increased rapidly after administration of LPS. Also, BAPC‐SME and BA‐SME could further promote the expression of IL‐10, thus enhancing its inhibitory effect on inflammation.

However, we found that BA could only inhibit the secretion of six inflammatory cytokines: TNF‐α, IL‐12, IL‐23, IFN‐β, RANTES, and MCP‐1. BA‐SME could also only inhibit the secretion of 10 inflammatory cytokines: TNF‐α, IL‐6, IFN‐γ, MCP‐1, IL‐17A, IL‐27, IL‐1α, MIG, IL‐23, and RANTES. The inhibitory effects of BAPC‐SME and BA‐SME on MCP‐1 were significantly higher than that of BA, and the inhibitory effects of BAPC‐SME on TNF‐α and IL‐6 were also significantly higher than those of BA‐SME. In conclusion, although different baicalein formulations inhibited systemic cytokine storm to some extent, BAPC‐SME could significantly improve the inhibitory activity of baicalein on systemic cytokine storm. This result also confirmed the previous conjecture that baicalein could be used in the treatment of cytokine storm.

### 3.7 Inhibitory effect on cytokines in mesenteric lymph nodes

**FIGURE 8:**
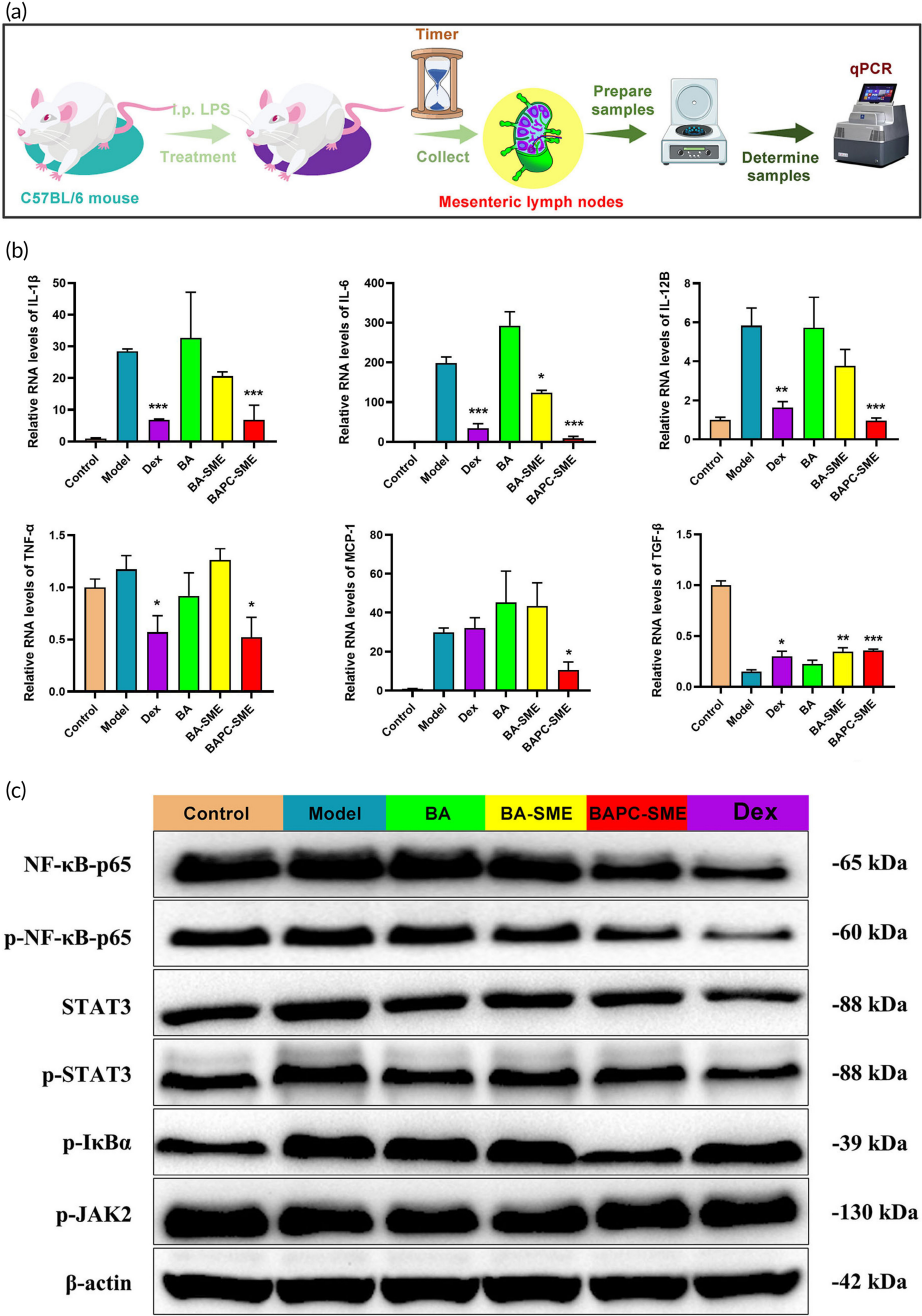
Inhibitory effect of BAPC‐SME on cytokines in mesenteric lymph nodes and its signal pathway. (a) The schematic of experimental process. (b) Relative transcript level of the mRNA of TNF‐α, IL‐1β, IL‐6, IL‐12B, MCP‐1, and TGF‐β in mesenteric lymph nodes of C57BL/6 mice after oral administration of BA, CBA‐SME, and BAPC‐SME following intraperitoneal injection of lipopolysaccharide for 1.5 h. The data are presented as the mean ± standard error of the mean (*n* = 3). *p < 0.05, **p < 0.01, ***p < 0.001 compared with model (Table S7). (c) Effects of BA, CBA‐SME, and BAPC‐SME on NF‐κB‐p65, p‐NF‐κB‐p65, STAT3, p‐STAT3, p‐IκBα, and p‐JAK2 protein expression in mesenteric lymph nodes

In this study, the qPCR technique was used to detect the mRNA expression of inflammatory cytokines in the mesenteric lymph nodes of mice in a systemic cytokine storm model, thus investigating whether BAPC‐SME could directly inhibit the mRNA expression of inflammatory cytokines in mesenteric lymph nodes through lymphatic transport. The mRNA expression of TNF‐α, IL‐1β, IL‐6, IL‐12B, and MCP‐1 in mesenteric lymph nodes of mice with cytokine storm decreased significantly after oral administration of BAPC‐SME (Figure 8b). Simultaneously, we found that both BAPC‐SME and BA‐SME could significantly promote the mRNA expression of the anti‐inflammatory cytokine TGF‐β in lymph nodes. In contrast, BA‐SME could only inhibit the expressions of and IL‐6, while BA showed no effect. The results suggested that baicalein in BAPC‐SME could directly act on the immune cells in lymph nodes through lymphatic targeted aggregation, thus inhibiting the expression of related inflammatory cytokines and enhancing the regulatory effect of baicalein on cytokine storm.

### 3.8 Inhibitory effect on NF‐κB and the STAT3 signal pathway

To explore the pharmacological mechanism by which BAPC‐SME inhibits the expression of inflammatory cytokines in the mesenteric lymph nodes of mice in the systemic cytokine storm model, the expression levels of related proteins of the NF‐κB and STAT3 signal pathway in mesenteric lymph nodes were determined via western blotting (Figure 8c). When compared with the model group, BAPC‐SME was found to reduce the expression of NF‐κB‐p65 and STAT3 protein in lymph nodes, as well as inhibit the phosphorylation of NF‐κB‐p65, IκBα, and STAT3. Simultaneously, the inhibitory effect of BAPC‐SME was stronger than that of BA and BA‐SME. These results suggested that BAPC‐SME could reduce the mRNA expression of downstream inflammatory cytokines by inhibiting the NF‐κB and STAT3 signaling pathways, thus exhibiting an inhibitory effect on systemic cytokine storm.

### 3.9 Inhibitory effect on local cytokine storm in the lung

**FIGURE 9:**
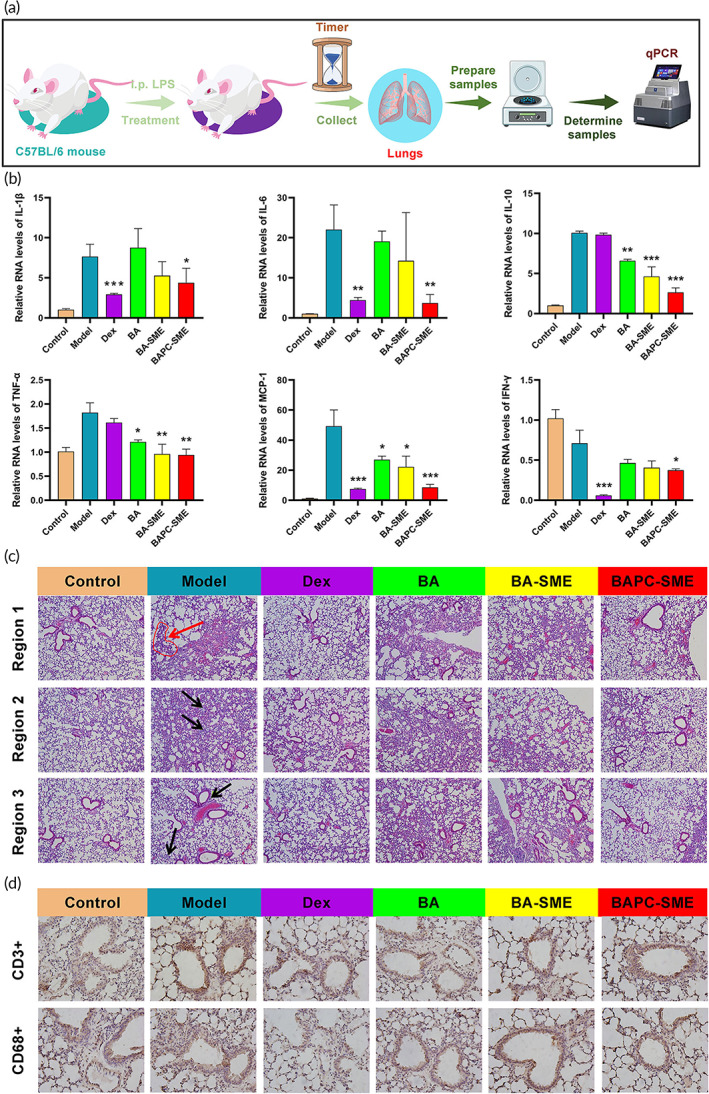
Inhibitory effect of BAPC‐SME on local cytokine storm in the lung. (a) The schematic of experimental process. (b) Relative mRNA level of TNF‐α, MCP‐1, IL‐1β, IL‐6, IL‐10, and IFN‐γ in the lung. The data are presented as the mean ± standard error of the mean (*n* = 4). *p < 0.05, **p < 0.01, ***p < 0.001 compared with model (Table S8). (c) The histopathological assays (magnification, ×100) in the lungs were performed at 6 h after LPS instillation. Alveolar collapse (black arrow), Inflammatory cell infiltration (red arrow). (d) Representative image (magnification, ×400) of immunohistochemically identified (brown) T cells and macrophages in lung tissues detected with murine T cells marker protein (CD3) and macrophage marker protein (CD68)

Six hours after intratracheal injection of LPS, the mRNA expression of TNF‐α, MCP‐1, IL‐1β, IL‐6, and IL‐10 in the lung tissue of mice was significantly increased (Figure 9b). Oral administration of BA and BA‐SME could significantly inhibit the expression of TNF‐α, MCP‐1, and IL‐10, and the inhibitory effects of BA‐SME on TNF‐α and IL‐10 were stronger than those of BA. Further, BAPC‐SME could significantly inhibit the level of TNF‐α, MCP‐1, IL‐1β, IL‐6, IFN‐γ, and IL‐10 in lung tissue, and increase the inhibitory effect of baicalein on related cytokines.

The above results showed that BAPC‐SME could significantly inhibit the occurrence of local pulmonary cytokine storm and effectively improve the inhibitory effect of baicalein on the expression of related inflammatory cytokines. Other than promoting the expression of IL‐10 in systemic cytokine storm, BAPC‐SME significantly inhibited the expression of IL‐10 in local pulmonary cytokine storm, which is beneficial to maintaining a low level of inflammatory response in the lung tissue, thus promoting the clearance of pathogens.

The pathological changes of lung tissue caused by local cytokine storm are shown in Figure 9c. The alveolar structure of lung tissue in the control group was regular and intact. The lung tissue injury among mice in the model group was obvious and included alveolar collapse, alveolar septal edema, inflammatory cell infiltration, alveolar wall thickening, telangiectasia, hyperemia, and partial bronchial destruction. Mucus and red blood cells appeared in the cavity. In the BA group, telangiectasia, hyperemia, partial alveolar wall thickening, and alveolar septal edema were observed. Part of the alveolar wall thickened in the BA‐SME group. In the BAPC‐SME group, there was a small amount of alveolar septal edema, and most of the alveolar structures were regular and complete. Part of the alveolar wall was observed to have thickened in the dexamethasone group. The results showed that baicalein significantly improved lung injury in model mice. Simultaneously, BAPC‐SME and BA‐SME could further enhance the improved effect of baicalein on lung tissue pathological injury.

The results of immunohistochemistry are shown in Figure 9d. Compared with the blank control group, there was significant infiltration by CD3 T lymphocytes in the lung tissue of mice in the model group, while both BA‐SME and BAPC‐SME could significantly inhibit infiltration by T cells. However, tissue infiltration by CD68 macrophages was not obvious.

## 4 DISCUSSION

### 4.1 Mesenteric lymph node tissue distribution, Caco‐2 cell uptake, and intracellular transport pathway of BAPC‐SME

In a previous study, our group successfully constructed a baicalein self‐microemulsion with the baicalein‐phospholipid complex as an intermediate carrier. BAPC‐SME improved the long‐term storage stability of baicalein self‐microemulsion and broke through the restriction on the construction of self‐microemulsion due to the low fat solubility of baicalein. Previous studies have also shown that BAPC‐SME did not only significantly increase the oral bioavailability of baicalein, but also increased the lymphatic transport of baicalein from 18.8% to 70.2%.^37^ In addition, after entering the intestinal lymphatic system, the drug would first be transferred to the mesenteric lymph nodes, and then enter the blood circulation through the collecting lymphatic vessels, thoracic ducts, and other structures.^8^, ^48^ Thus, immunomodulators can act directly on the immune cells of mesenteric lymph nodes to exhibit anti‐inflammatory and immunosuppressive effects through lymphatic transport. Therefore, based on the results of previous studies, DiR and Cou‐6 were used to label BAPC‐SME in the present study. A small animal imaging device and flow cytometry were used to detect the concentration of BAPC‐SME in mesenteric lymph nodes. The results further proved that BAPC‐SME could target baicalein aggregation in mesenteric lymph nodes via lymphatic transport, which is beneficial in enabling baicalein to act directly on immune cells in lymph nodes to play an immunomodulatory role.

In addition, we speculated that BAPC‐SME could not only promote the oral absorption of baicalein by increasing its solubility and dissolution, but also enhanced the absorption and lymphatic transport of baicalein through direct uptake by intestinal epithelial cells. Therefore, the uptake of BAPC‐SME in intestinal epithelial cells was investigated using a Caco‐2 cell model and its cellular uptake and the intracellular transport pathways involved were discussed. Based on the experimental study of this paper and the related literature,^49^, ^50^ the specific process of uptake and secretion of BAPC‐SME by intestinal epithelial cells was speculated as follows. First, BAPC‐SME would enter intestinal epithelial cells through a lipid valve and clathrin‐mediated endocytosis. Then, the intracellular vesicles enclosing BAPC‐SME are transferred to early inclusions. After a series of intracellular transformations, the early inclusion body becomes a late inclusion body and fuses with other cellular components to form a lysosome. Finally, the lysosome might fuse with a chylomicron in the endoplasmic reticulum or Golgi matrix and undergo transfer to the cell plasma membrane, thereby exiting the cell.

### 4.2 Inhibitory effect of BAPC‐SME on systemic cytokine storm

Some studies have shown that the levels of characteristic cytokines such as TNF‐α, IL‐6, IL‐1β, IL‐1α, IL‐12, IL‐17, CXCL1, CXCL2, MCP‐1, IL‐8, CXCL10, GM‐CSF, M‐CSF, and G‐CSF in patients with septicemia are significantly higher than those in normal volunteers.^12^ The occurrence of cytokine storm is not only a significant clinical feature of septicemia, but also an important cause of the systemic inflammatory response. Therefore, septicemia‐related cytokine storm can be used as a representative disease in the study of systemic cytokine storm caused by infection. Based on the septicemia model induced by LPS, systemic cytokine storm was induced by intraperitoneal injection of LPS in this study. Also, the dynamic changes in inflammatory cytokine levels in the plasma of mice within 24 h after the establishment of the model were examined and analyzed for the first time. This approach to modeling cytokine storm was simple and effective, and had important reference significance for related research of drugs used in the treatment of cytokine storm.

A previous study reported that the levels of IL‐1β, IL‐6, IL‐8, IL‐12, IP‐10, MCP‐1, and IFN‐ γ in patients with SARS infection increased significantly.^51^ MERS‐CoV infection could also induce an increase in the concentration of IL‐15, IL‐17, IFN‐γ, and TNF‐α.^52^ In addition, some studies found that the levels of IL‐2, IL‐4, IL‐6, IL‐7, IL‐10, IP‐10, MCP‐1, TNF‐α, MIP‐1α, IFN‐γ, and G‐CSF in patients with severe COVID‐19 infection were significantly higher than those in patients with mild and moderate infection.^13^, ^53^, ^54^ The present study confirmed that oral administration of BAPC‐SME could significantly inhibit the increase in the plasma levels of 14 inflammatory cytokines: TNF‐α, IL‐6, IFN‐γ, MCP‐1, IL‐17A, IL‐27, IL‐1α, GM‐CSF, MIG, IFN‐β, IL‐12, MIP‐3α, IL‐23, and RANTES. On the other hand, BAPC‐SME also targeted the aggregation of baicalein in the mesenteric lymph nodes through lymphatic transport and directly acted on the immune cells in the lymph nodes, thus inhibiting the mRNA expression of inflammatory cytokines such as TNF‐α, IL‐1β, IL‐6, IL‐12B, and MCP‐1. This covers the types of cytokines associated with cytokine storm among most patients with MERS‐CoV, SARS, and COVID‐19, suggesting that BAPC‐SME has the potential to regulate systemic cytokine storm induced by most bacterial and viral infections.

### 4.3 Inhibitory effect of BAPC‐SME on the NF‐κB and STAT3 signal pathway

The NF‐κB nuclear factor family is composed of c‐Rel, Rel A (p65), Rel B, p50/p105, and p52/p100. In most inactivated cells, NF‐κB binds to the inhibitor IκB in an inactive state. When cells are activated by extracellular stimuli such as pathogens, cytokines, and stress signals, IκB is phosphorylated, ubiquitinated, and degraded by protease, after which NF‐κB is released and phosphorylated. Phosphorylated NF‐κB is transferred to the nucleus to regulate a variety of targeted gene transcriptions in different cells, thus controlling the expression of proinflammatory cytokines, chemokines, immune receptors, and cell surface adhesion molecules.^55^, ^56^ Signal transducer and activator of transcription (STAT) can be phosphorylated and dimerized by Janus kinase (JAK) and transferred into the nucleus to regulate the expression of related genes; this signal pathway is called the JAK/STAT signal pathway. JAK/STAT protein is widely expressed and may play a role in regulating and maintaining a series of basic biological processes, including apoptosis, proliferation, immune response, and inflammation.^57^, ^58^

In the present study, western blotting showed that BAPC‐SME could reduce the expression of NF‐κB‐p65 and STAT3 protein, and inhibit the phosphorylation of NF‐κB‐p65, IκBα, and STAT3 in the mesenteric lymph nodes of mice, thus blocking the activation of the NF‐κB and STAT3 signal pathway and reducing the downstream mRNA expression of TNF‐α, IL‐1β, IL‐6, IL‐12B, and MCP‐1.

### 4.4 BAPC‐SME inhibited pathological injury caused by local cytokine storm in the lung

Cytokine storm usually starts with acute inflammation of local tissue and then spreads to the whole body through the systemic circulation. Acute lung injury (ALI) is a common consequence of cytokine storm in the alveolar environment or systemic circulation, usually associated with infection of the lungs or other organs.^59^ For example, SARS coronavirus and influenza virus infection could cause ALI or its more severe form, acute respiratory distress syndrome. The most typical example of cytokine storm occurs in the process of severe pulmonary infection, in which local inflammation overflows into the systemic circulation, resulting in systemic septicemia and systemic cytokine storm. Typical symptoms include persistent hypotension, hyperthermia or hypothermia, leukocytosis, and recurrent thrombocytopenia.^60^ Viral, bacterial, and fungal pulmonary infections could all cause systemic cytokine storm, and these causes are difficult to distinguish clinically. In some cases, persistent lung tissue damage without severe microbial infection is also associated with the clinical manifestations of cytokine storm. In addition to pulmonary infections, cytokine storm also results from severe infections in the gastrointestinal tract, urinary tract, central nervous system, skin, joint spaces, and other tissue.

In the present study, the therapeutic effects of different baicalein formulations on lung injury caused by LPS‐induced cytokine storm were evaluated in vivo. The results showed that oral administration of BAPC‐SME could significantly increase the inhibitory effect of baicalein on six kinds of cytokines in lung tissue: TNF‐α, MCP‐1, IL‐1β, IL‐6, IFN‐γ, and IL‐10. BAPC‐SME also significantly improved pathological injury and T lymphocyte infiltration in lung tissue.

## 5 CONCLUSIONS

In the present study, baicalein self‐microemulsion with phospholipid complex as an intermediate carrier could significantly promote the targeted aggregation of baicalein in mesenteric lymph nodes, which was beneficial for the direct inhibition of the secretion of inflammatory cytokines in lymph nodes by baicalein, thus enhancing the therapeutic effect of baicalein on cytokine storm. In vivo studies have shown that oral administration of BAPC‐SME effectively inhibits the expression of related inflammatory cytokines in systemic cytokine storm and local pulmonary cytokine storm, and significantly improves tissue damage caused by cytokine storm. The present study did not only provide a new lymphatic targeted drug delivery strategy for the treatment of infection‐related cytokine storm, but also laid a foundation for the clinical application of baicalein.

## AUTHOR CONTRIBUTIONS

Hengfeng Liao designed the research, wrote the manuscript, carried out the experiments, and performed the data analysis. Jun Ye, Yue Gao, Chunfang Lian, Lu Liu, Xiaoyan Xu, Yu Feng, Yanfang Yang, Yuqi Yang, Qiqi Shen, Lili Gao, and Zhihua Liu participated in the experiments. Yuling Liu and Jun Ye revised the manuscript. Yuling Liu supervised and managed the project. All authors have read and approved the final manuscript.

## CONFLICT OF INTEREST

The authors declare no conflict of interest.

## Supporting information


**Table S1** Program of gradient elution.
**Table S2** The content and related substance of BAPC‐SME at 4°C and 25°C for sixth month.
**Table S3**. Minimum inhibitory concentrations (MICs) of baicalein (BA), baicalin (BG), BAPC‐SME, and levofloxacin.
**Table S4**. The establishment of systemic cytokine storm model. The data are presented as the minimum value/median/maximum value (pg/ml, *n* = 4–5).
**Table S5**. Mean plasma concentration of IL‐6 in C57BL/6 mice after oral administration of different doses of BAPC‐SME following intraperitoneal injection of lipopolysaccharide (5 mg/kg). The data are presented as the mean ± standard error of the mean (*n* = 5).
**Table S6**. Mean plasma concentration of cytokine in C57BL/6 mice after oral administration of BA, BA‐SME, and BAPC‐SME following the intraperitoneal injection of lipopolysaccharide. The data are presented as the minimum value/median/maximum value (pg/ml, *n* = 4–6).
**Table S7**. Relative transcript level of the mRNA of TNF‐α, IL‐1β, IL‐6, IL‐12B, MCP‐1, and TGF‐β in mesenteric lymph nodes of C57BL/6 mice after oral administration of BA, CBA‐SME, and BAPC‐SME following intraperitoneal injection of lipopolysaccharide for 1.5 h. The data are presented as the mean ± standard error of the mean (*n* = 3).
**Table S8**. Relative mRNA level of TNF‐α, MCP‐1, IL‐1β, IL‐6, IL‐10, and IFN‐γ in the lung. The data are presented as the mean ± standard error of the mean (*n* = 4).
**Figure S1**. IR spectrum (A), X‐ray diffractometry spectra (B), and DSC thermograms (C) of BA, PC, BAPC and the physical mixture of BA and PC.
**Figure S2** The effect of BAPC‐SME on protein and mRNA expression of coronavirus (A–B) and influenza virus (C,D).

## Data Availability

The data are available in the main manuscript. Additional information files are available from the corresponding author by request.
