# Supplementary material for: Baicalein self‐microemulsion based on drug–phospholipid complex for the alleviation of cytokine storm
Source: Bioeng Transl Med. 2022 Jun 20;8(1):e10357. doi: 10.1002/btm2.10357 (PMC9842031; doi:10.1002/btm2.10357)
Supplement: Supplementary file 1 — Table S1 Program of gradient elution. Table S2 The content and related substance of BAPC‐SME at 4°C and 25°C for sixth month. Table S3. Minimum inhibitory concentrations (MICs) of baicalein (BA), baicalin (BG), BAPC‐SME, and levofloxacin. Table S4. The establishment of systemic cytokine storm model. The data are presented as the minimum value/median/maximum value (pg/ml, n = 4–5). Table S5. Mean plasma concentration of IL‐6 in C57BL/6 mice after oral administration of different doses of BAPC‐SME following intraperitoneal injection of lipopolysaccharide (5 mg/kg). The data are presented as the mean ± standard error of the mean (n = 5). Table S6. Mean plasma concentration of cytokine in C57BL/6 mice after oral administration of BA, BA‐SME, and BAPC‐SME following the intraperitoneal injection of lipopolysaccharide. The data are presented as the minimum value/median/maximum value (pg/ml, n = 4–6). Table S7. Relative transcript level of the mRNA of TNF‐α, IL‐1β, IL‐6, IL‐12B, MCP‐1, and TGF‐β in mesenteric lymph nodes of C57BL/6 mice after oral administration of BA, CBA‐SME, and BAPC‐SME following intraperitoneal injection of lipopolysaccharide for 1.5 h. The data are presented as the mean ± standard error of the mean (n = 3). Table S8. Relative mRNA level of TNF‐α, MCP‐1, IL‐1β, IL‐6, IL‐10, and IFN‐γ in the lung. The data are presented as the mean ± standard error of the mean (n = 4). Figure S1. IR spectrum (A), X‐ray diffractometry spectra (B), and DSC thermograms (C) of BA, PC, BAPC and the physical mixture of BA and PC. Figure S2 The effect of BAPC‐SME on protein and mRNA expression of coronavirus (A–B) and influenza virus (C,D). [file BTM2-8-e10357-s001.doc]

Supporting Information

Baicalein self-microemulsion based on drug–phospholipid complex for the alleviation of cytokine storm

Hengfeng Liao, Jun Ye, Yue Gao, Chunfang Lian, Lu Liu, Xiaoyan Xu, Yu Feng, Yanfang Yang, Yuqi Yang, Qiqi Shen, Lili Gao, Zhihua Liu, Yuling Liu [[1]](#footnote-2)*

# Method

## Characterization of BAPC

**X-ray Diffraction (XRD)**

The physical states of BA, phospholipid (PC), BAPC and the physical mixture of BA and PC were determined by using an X-ray diffractometer (Bruker D8, Bruker, Germany) with a tube voltage of 40 kV and a current of 40 mA. All samples were scanned over a range of 2θ angles from 3 ° to 60 °.

**Fourier transform infrared spectroscopy (FT-IR)**

The infrared spectra of BA, PC, BAPC and the physical mixture of BA and PC were scanned in the 400~4000 cm^-1^ range using iS10 FT-IR spectrometer (Thermo Nicolet Corporation, America). The resolution of the spectrometer was 4 cm^-1^, and the signal-to-manic ratio was 50000.

**Differential Scanning Calorimetry (DSC)**

A differential scanning calorimeter (DSC 6200, Seiko Instruments Inc, Japan) was employed to take thermograms of BA, PC, BAPC and the physical mixture of BA and PC. The samples were heated from 25 ℃ to 350 ℃ at a rate of 10 ℃/min with the nitrogen flow rate of 60 mL/min.

## Storage stability of BAPC-SME

The BAPC-SME was stored at 4 ℃ and 25 ℃ for 6 months. And the changes of its content and related substances were determined at 0, 2, 4 and 6 months.

The related substances of BAPC-SME was determined using the Agilent 1260 HPLC system from Agilent Technologies (Santa Clara, CA, USA), equipped with an ES Industries® FluoroSep-RP Phenyl (25 cm×4.6 mm，5 μm), at room temperature. The mobile phase was composed of methanol, acetonitrile and 0.1% phosphoric acid. Linear gradient elution (Table S1) was used. The flow rate was 1.0 mL/min. A 10 μL volume was injected into the column and the wavelength for detection was 275 nm.

Table S1. Program of gradient elution

| Time (min) | Percentage of each eluent (%) (v/v) | | |
| --- | --- | --- | --- |
| 0.1% [phosphoricacid](https://fanyi.so.com/?src=onebox#phosphoric%20acid) | Methanol | Acetonitrile |
| 0  5  7  15  25  40  41  46 | 85  85  40  40  42  42  85  85 | 15  15  60  60  0  0  15  15 | 0  0  0  0  60  60  0  0 |

## In vitro antibacterial activity

The minimum inhibitory concentrations (MICs) of baicalein (BA), baicalin (BG), and BAPC-SME were determined using the agar dilution method described by the Clinical Laboratory Standards Institute. The MIC was defined as the lowest concentration that prevented the visible growth of the bacteria. Stock solutions of BA, BG, and levofloxacin (positive control) were prepared in 0.2% dimethyl sulfoxide and at concentrations of 1920 μg/mL.

We prepared intermediate (15×) antimicrobial agent solutions by making serial twofold dilutions. Then, we added 1 mL of the antimicrobial solution to 14 mL of molten agar. The final concentrations of the compounds ranged from 0.03 to 128 μg/mL. We adjusted the turbidity of the actively growing broth with sterile saline, and compared the inoculum tube and the 0.5 McFarland standard to determine the inoculum size, which was found to be 10000 colony forming units per spot. Culture plates were incubated at 35±2°C for 18 h and the MICs were then recorded.

## In vitro antiviral study

BAPC-SME was diluted to different concentrations with cell culture medium. H460 cells were cultured with 5% CO_2_ in a 96-well culture plate at 37°C. After 24 h, the cells were infected with coronavirus, and the maintenance media containing different concentrations of BAPC-SME or ribavirin (positive control) were added. Maintenance medium without a drug was added to the blank control group. When the cytopathic effect (CPE) of the blank control group reached 4+, the CPE of each group was observed. The median toxic concentration and half-maximal inhibitory concentration (IC_50_) of the sample were calculated using the Reed–Muench method.

H460 cells (1.5×10^5^/mL) were seeded in 12-well plates and cultured overnight. The cells were infected with human coronavirus OC43 (HCoV-OC43), and different concentrations of drug solutions and ribavirin were added. After the cells were cultured with 5% CO_2_ at 37°C for 24 h, total RNA was extracted using a RNeasy Mini Kit and the mRNA expression level of the nucleoprotein (NP) of HCoV-OC43 was detected via quantitative polymerase chain reaction (qPCR) assay. In addition, total cell protein was extracted after the cells were cultured with 5% CO_2_ at 37°C for 48 h, and the expression level of the NP of HCoV-OC43 in the cells was detected via western blotting.

Madin–Darby Canine Kidney cells (5×10^5^/mL) were seeded in 24-well plates and cultured overnight. The cells were infected with influenza virus A, and different concentrations of BAPC-SME or ribavirin were added. After the cells were cultured with 5% CO_2_ at 37°C for 24 h, total RNA was extracted using a RNeasy Mini Kit and the mRNA expression level of the M2 protein of influenza virus was detected via qPCR assay. Total cell protein was extracted and the expression level of the M2 protein of influenza virus was detected via western blotting.

# Results

## Characterization of BAPC

**XRD**

According to Figure S1B, baicalein has obvious crystal diffraction peak, while phospholipid is amorphous, but when baicalein and phospholipid form drug-phospholipid complex, the crystal diffraction peak of baicalein disappears, indicating that baicalein exists in amorphous form in phospholipid complex. However, the crystal diffraction peak of baicalein can still be seen in the physical mixture of baicalein and phospholipid, indicating that baicalein is still crystalline in the physical mixture, which also confirms the formation of baicalein phospholipid complex.

**FT-IR**

According to Figure S1A, the 3413.27 cm^-1^ peak is the stretching vibration peak of baicalein phenolic hydroxyl (- OH), and the 1656.79, 1618.24 and 1585.27 cm^-1^ peak is the vibration peak of the benzene ring structure of baicalein molecule. The stretching vibrations peak of the phosphorus-oxygen double bonds at the polar end of phospholipids is at 1243.24 cm^-1^, while the characteristic peaks of the non-polar fat chain of phospholipids are 2927.28 and 2854.69 cm^-1^. At the same time, it can be seen from Figure S1A that the spectrum of the physical mixture is the superposition of the IR spectrum of baicalein and phospholipid. The corresponding characteristic peaks of baicalein and phospholipid can also be found in the spectrum of the physical mixture, and their intensity and peak position have no significant change. It shows that there is no new chemical bond, hydrogen bond or van der Waals force between baicalein and phospholipids in the physical mixture. However, in the spectrum of baicalein phospholipid complex, the stretching vibration peak of baicalein phenolic hydroxyl group (- OH) disappears, while the stretching vibration peak of baicalein benzene ring structure and phosphorus-oxygen double bond at the polar end of phospholipid also change slightly, indicating that there may be a new hydrogen bond between the phenolic hydroxyl group of baicalein and the phosphorus-oxygen double bond of phospholipid.

**DSC**

In Figure S1C, it is obvious that BA has obvious endothermic peak at 270.1 ℃ and PC has no obvious endothermic peak. The physical mixture has small endothermic peak at 192.8 ℃ and 228.3 ℃ without BA characteristic endothermic peak. And BAPC has small endothermic peak at 169.7 ℃, but there is no BA characteristic endothermic peak.





Figure S1. IR spectrum (A), X-ray diffractometry spectra (B) and DSC thermograms (C) of BA, PC, BAPC and the physical mixture of BA and PC.

## Storage stability of BAPC-SME

In addition, as shown in Table S2, the concentration of baicalein in BAPC-SME stored at 4 ℃ and 25 ℃ for 6 months was 98.17% and 94.73% of the initial value, respectively. And the related substances of BAPC-SME stored at 4 ℃ and 25 ℃ for 6 months were 0.79% and 1.28%, respectively. The results show that BAPC-SME can be stored stably at 4 ℃ and 25 ℃.

Table S2 The content and related substance of BAPC-SME at 4 ℃ and 25 ℃ for sixth month

| **Tempetarature** | **Time (months)** | **Content (%)** | **Related substance (%)** |
| --- | --- | --- | --- |
| 4 ℃ | 0 | 100.00 | 0.77 |
|  | 2 | 97.84 | 1.00 |
|  | 4 | 97.45 | 0.88 |
|  | 6 | 98.17 | 0.79 |
| 25 ℃ | 0 | 100.00 | 0.77 |
|  | 2 | 98.49 | 1.42 |
|  | 4 | 95.11 | 1.17 |
|  | 6 | 94.73 | 1.28 |

## In vitro antibacterial activity

Most cytokine storms are caused by pathogenic bacteria or viral infections. Therefore, investigation of the direct antibacterial activity of BA, BG, and BAPC-SME is of great significance in evaluating the use of baicalein for the treatment of infection-related cytokine storm. Baicalin had no inhibitory effect on most strains and BAPC-SME could enhance the inhibitory activity of baicalein against *Staphylococcus epidermidis*, *Staphylococcus aureus*, *Enterococcus faecalis*, *Enterococcus faecium*, *Escherichia coli*, *Klebsiella pneumoniae*, *Pseudomonas aeruginosa*, and Acinetobacter *baumannii* (Table S3). In addition, BAPC-SME had the best inhibitory activity against *Staphylococcus epidermidis*, *Staphylococcus aureus*, and *Acinetobacter baumannii*, and its MIC was as low as 64 μg/mL.

Table S3. Minimum inhibitory concentrations (MICs) of baicalein (BA), baicalin (BG), BAPC-SME, and levofloxacin

| Bacterial strain | MIC (μg/mL) | | | |
| --- | --- | --- | --- | --- |
|  | BA | BG | BAPC-SME | Levofloxacin |
| *Staphylococcus epidermidis* | 128 | >512 | 64 | 2 |
| *Staphylococcus aureus* | 128 | >512 | 64 | 0.12 |
| *Enterococcus faecalis* | 1024 | >512 | 512 | 0.5 |
| *Enterococcus faecium* | 1024 | >512 | 256 | 64 |
| *Escherichia coli* | 512 | >512 | 256 | 8 |
| *Klebsiella pneumoniae* | 512 | >512 | 256 | 0.5 |
| *Pseudomonas aeruginosa* | 512 | >512 | 256 | 0.5 |
| *Acinetobacter baumannii* | 128 | 512 | 64 | 0.12 |
| *Enterobacter cloacae* | 128 | >512 | 128 | 0.06 |
| *Enterobacter aerogenes* | 256 | >512 | 256 | 0.06 |
| *Serratia marcescens* | 256 | >512 | 256 | 0.12 |
| *Citrobacter freundii* | 256 | >512 | 256 | ≤0.03 |
| *Proteus mirabilis* | 128 | >512 | 128 | ≤0.03 |
| *Pseudomonas maltophilia* | 64 | >512 | 128 | 2 |
| *Shigella flexneri* | 256 | >512 | 256 | ≤0.03 |

## In vitro antiviral study

Previous studies have reported that baicalein, as the main active substance in *Scutellaria baicalensis* Georgi, has antiviral activity against herpesviruses, some human adenoviruses, respiratory syncytial viruses, and other viruses^[1, 2]^. Therefore, in the present study, the CPE method was used to investigate the in vitro activity of baicalein and its metabolite baicalin against coronavirus, respiratory syncytial virus, and influenza virus at the cellular level with ribavirin as the positive control drug. The IC_50_ of BAPC-SME against coronavirus (HCoV-OC43) was 4.94 μg/mL, indicating that it had certain anti-coronavirus activity.

Simultaneously, the results of western blot experiments and qPCR (Figures S2A and S2B) showed that BAPC-SME could inhibit the mRNA expression of the NP of HCoV-OC43 both at concentrations of 2 and 1 μg/mL, with an inhibition rate of about 70%, while BAPC-SME directly inhibited the expression of the NP of HCoV-OC43 only at 2 μg/mL. In addition, BAPC-SME had a slightly inhibitory effect on the expression of the M2 protein of seasonal influenza strain A (H3N2) at concentrations of 2, 1, and 0.5 μg/mL (Figures S2C). However, BAPC-SME could significantly inhibit the mRNA expression of the M2 protein of the influenza virus at a concentration of 2 μg/mL (Figures S2D), with an inhibition rate of 57.9%.


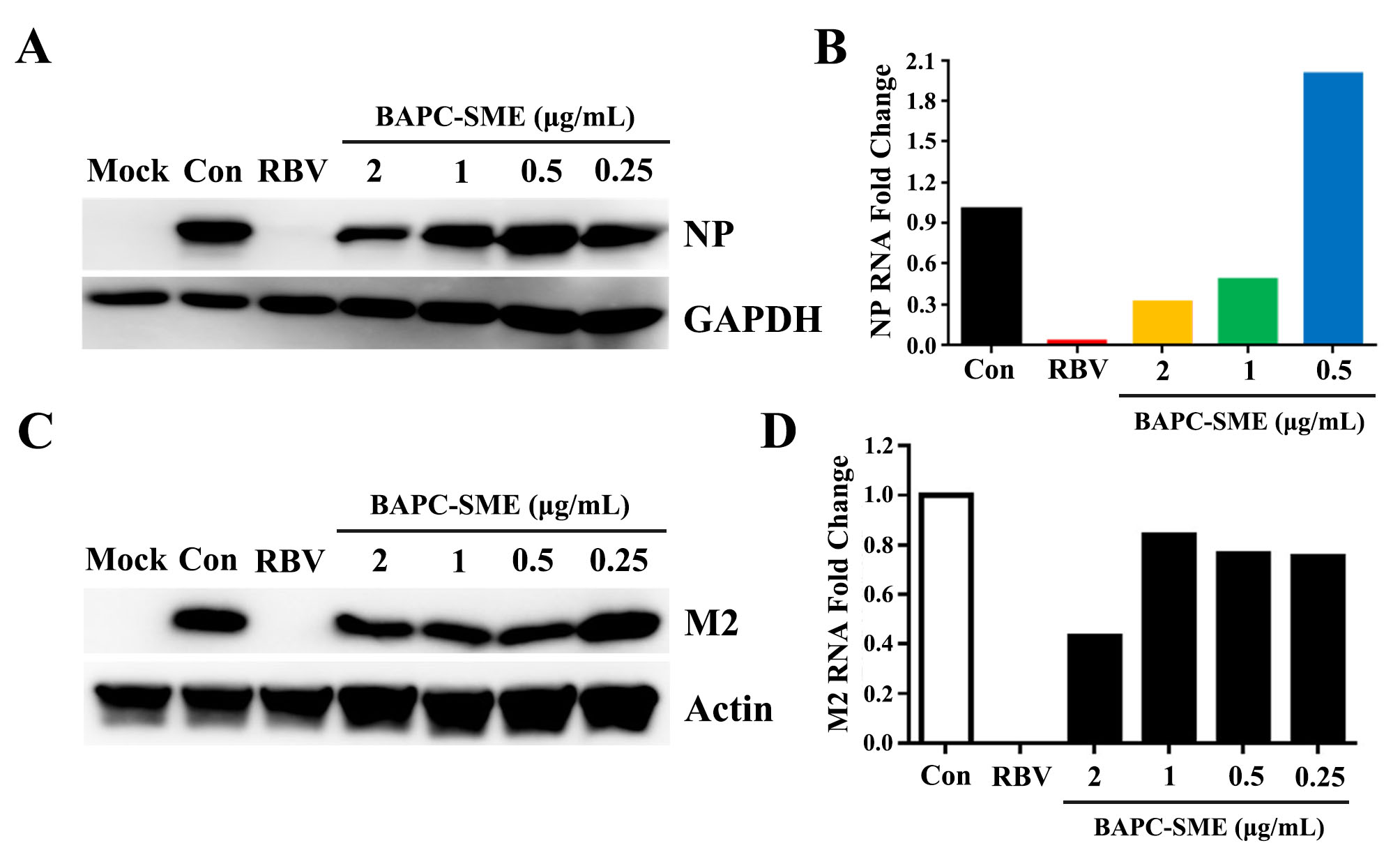


Figure S2 The effect of BAPC-SME on protein and mRNA expression of coronavirus (A–B) and influenza virus (C–D).

Table S4. The establishment of systemic cytokine storm model. The data are presented as the minimum value/median/maximum value (pg/mL, n=4~5).

| Time (h) | 0 | 1.5 | 4 | 6 | 8 | 12 | 24 |
| --- | --- | --- | --- | --- | --- | --- | --- |
| IL-1α | 4.79/5.60/6.00 | 16.47/18.51/25.43 | 31.62/37.07/66.86 | 11.61/34.57/68.18 | 8.14/33.84/70.20 | 24.80/25.27/33.10 | 13.14/15.69/21.47 |
| IFN-γ | 5.89/9.41/15.52 | 9.68/15.45/19.28 | 185.16/297.37/1284.15 | 1126.96/1710.64/2720.15 | 435.24/1712.25/3486.75 | 213.05/320.18/537.46 | 12.02/14.09/14.32 |
| TNF-α | 13.83/14.78/37.65 | 5582.05/9012.23/19344.1 | 479.90/548.75/776.77 | 149.70/362.89/457.64 | 218.51/262.63/289.96 | 108.62/136.52/162.80 | 43.72/59.60/106.62 |
| MCP-1 | 36.22/38.52/68.23 | 6877.52/9227.64/9324.86 | 10093.95/13202.82/17556.16 | 7113.72/13571.05/17231.50 | 3202.95/5298.39/7926.24 | 830.45/1010.58/1523.41 | 366.72/487.53/696.84 |
| IL-12p70 | 3.04/6.20/14.63 | 12.60/17.55/23.48 | 17.02/23.97/70.76 | 5.53/12.80/23.75 | 4.48/7.22/38.82 | 3.36/4.41/5.49 | 2.54/3.19/8.20 |
| IL-10 | 8.40/37.37/126.10 | 726.99/845.58/1004.90 | 234.59/295.44/371.60 | 114.43/181.28/360.08 | 19.64/84.28/127.43 | 84.15/110.56/161.90 | 147.99/158.14/161.90 |
| IL-27 | 84.49/486.94/589.74 | 222.01/258.02/652.81 | 1207.58/3313.43/5221.81 | 239.46/2538.77/3851.61 | 941.01/1206.10/2716.58 | 78.61/126.45/206.61 | 28.4/294.905/528.66 |
| IL-17A | 4.78/10.33/17.13 | 8.16/11.53/14.91 | 23.06/25.21/26.62 | 41.72/59.295/65.43 | 22.09/83.60/88.67 | 45.72/66.57/101.25 | 11.81/15.60/24.37 |
| IFN-β | 80.70/113.37/307.24 | 861.50/1400.25/3060.41 | 99.17/135.01/611.47 | 87.85/214.32/490.72 | 119.82/166.09/254.16 | 78.94/104.30/194.14 | 73.24/97.04/195.41 |
| GM-CSF | 1.75/16.57/21.43 | 11.51/16.62/20.33 | 19.58/20.85/30.01 | 7.60/10.39/49.83 | 4.52/6.26/11.19 | 3.19/7.78/17.64 | 0.22/3.02/8.89 |
| MIP-1β | 1514.55/1966.83/2335.43 | 12820.96/25796.86/30623.91 | 14409.17/17734.96/28214.34 | 5016.20/7625.68/9496.02 | 2050.23/2538.64/3067.60 | 409.55/873.78/1478.12 | 100.70/129.51/284.13 |
| MIG | 24.45/71.55/293.09 | 142.32/398.16/1495.30 | 6378.74/9130.87/10881.50 | 13678.50/14959.54/17395.06 | 12062.06/15063.58/17199.56 | 9196.77/11862.89/16415.73 | 2218.72/2655.14/4132.17 |
| IL-4 | 46.06/77.23/527.44 | 867.78/2147.33/3153.58 | 743.82/934.49/1084.01 | 241.83/400.59/935.10 | 113.43/139.00/206.56 | 25.83/49.04/122.09 | 0.09/9.41/11.80 |
| MIP-1α | 109.46/140.04/192.67 | 122.50/185.74/572.32 | 2397.77/3840.64/4125.93 | 4891.05/5909.16/6906.58 | 4731.38/5946.12/6318.37 | 3451.39/4465.35/6523.69 | 839.34/982.85/1557.10 |
| IL6 | 1566.27/1871.81/3698.22 | 58468.54/82482.87/113458.52 | 133415.83/158221.50/170220.41 | 44156.73/59579.14/109769.19 | 20278.88/33712.58/55832.35 | 8064.21/28742.08/47827.70 | 125.78/157.01/281.24 |

Table S5. Mean plasma concentration of IL-6 in C57BL/6 mice after oral administration of different doses of BAPC-SME following intraperitoneal injection of lipopolysaccharide (5 mg/kg). The data are presented as the mean ± standard error of the mean (n=5).

| Group | IL-6 (ng/mL) |
| --- | --- |
| Control | 1.1±0.1 |
| Model | 128.8±14.5 |
| Dex | 32.4±6.0 |
| BAPC-SME-20 | 123.5±13.1 |
| BAPC-SME-40 | 117.0±8.4 |
| BAPC-SME-80 | 58.8±19.9 |
| BAPC-SME-160 | 98.3±9.5 |

Table S6. Mean plasma concentration of cytokine in C57BL/6 mice after oral administration of BA, BA-SME, and BAPC-SME following the intraperitoneal injection of lipopolysaccharide. The data are presented as the minimum value/median/maximum value (pg/mL, n=4~6).

| Group | Control | Model | Dex | BA | BA-SME | BAPC-SME |
| --- | --- | --- | --- | --- | --- | --- |
| IL-1α | 3.47/4.65/6.36 | 44.30/48.03/58.18 | 13.4/ 21.06/ 42.23 | 26.14/ 40.94/ 54.09 | 20.81/ 29.53/ 35.20 | 19.19/ 32.56/ 44.30 |
| IL-1β | 23.24/25.46/31.78 | 98.63/ 101.57/ 113.63 | 82.47/ 94.60/ 199.87 | 49.40/ 89.31/ 93.38 | 83.78/ 86.88/ 111.88 | 82.29/ 88.53/ 109.16 |
| IL-6(ng/mL) | N/A | 53.71/ 62.68/ 78.98 | N/A | 39.67/ 69.29/ 81.74 | 27.88/ 43.38/ 59.74 | 5.43/ 12.73/ 18.89 |
| IL-10 | 35.62/ 85.38/ 117.32 | 218.75/ 413.63/ 536.29 | 670.27/ 1073.30/ 1145.76 | 499.51/ 533.96/ 595.42 | 465.12/ 712.53/ 1027.46 | 573.89/ 633.07/ 1017.57 |
| IL-12 | 0.44/ 2.03/ 3.02 | 68.47/ 86.88/ 107.37 | 46.30/ 56.88/ 66.87 | 18.02/ 69.64/ 81.81 | 50.30/ 65.12/ 85.42 | 50.36/ 58.09/ 76.29 |
| IL-17A | 3.40/ 7.14/ 14.03 | 18.29/ 27.27/ 55.89 | 1.87/ 2.87/ 8.33 | 19.39/ 28.43/ 31.80 | 4.30/ 9.14/ 17.10 | 4.09/ 8.22/ 15.37 |
| IL-23 | 49.88/ 84.28/ 118.77 | 382.30/ 463.11/ 751.32 | 290.42/ 427.00/ 534.66 | 300.09/ 326.05/  352.78 | 327.11/ 356.28/ 385.41 | 286.48/ 366.72/ 434.75 |
| IL-27 | 78.43/ 125.68/ 198.26 | 202.48/ 380.76/ 574.00 | 0.00/ 70.19/ 223.45 | 131.00/ 352.19/ 687.08 | 68.99/ 165.44/ 307.43 | 90.18/ 181.92/ 354.30 |
| TNF-α | N/A | 11270.77/ 14024.63/ 16547.64 | N/A | 3840.34/ 10024.42/ 15899.76 | 2319.11/ 3150.64/ 5435.24 | 1064.81/ 1170.70/ 1376.16 |
| IFN-β | N/A | 255.09/ 283.98/ 400.43 | 202.25/ 235.50/ 307.48 | 100.97/ 217.14/ 245.79 | 198.76/ 206.58/ 325.81 | 175.74/ 186.32/ 240.35 |
| IFN-γ | 3.09/ 4.58/ 5.99 | 746.27/ 929.17/ 1439.87 | 9.43/ 31.24/ 52.74 | 321.32/ 698.99/ 1286.56 | 57.62/ 138.14/ 352.58 | 30.08/ 126.87/ 435.28 |
| MCP-1 | 38.77/ 58.33/ 78.34 | 8973.64/ 10478.08/ 17585.93 | 382.12/ 504.97/ 769.84 | 2856.4/ 7368.99/ 11714.05 | 2231.81/ 2974.58/ 4187.70 | 1466.09/ 3062.56/ 4908.69 |
| MIP-3α | 23.56/ 43.96/ 79.14 | 2345.97/ 3412.43/ 4134.39 | 937.97/ 1524.87/ 2718.14 | 1110.83/ 4220.57/ 6249.35 | 1923.54/ 3307.92/ 9551.93 | 954.67/ 1571.05/ 3212.39 |
| MIG | 525.32/ 736.37/ 1012.15 | 12365.92/ 17757.44/ 44165.04 | 1728.71/ 3447.13/ 6089.26 | 5147.87/ 18011.9/ 30155.38 | 5419.78/ 6460.36/ 13189.3 | 5382.51/ 12858.99/ 14009.81 |
| GM-CSF | 1.71/ 6.72/ 9.21 | 8.65/ 10.55/ 17.19 | 1.36/ 4.19/ 11.5 | 5.08/ 9.72/ 14.43 | 2.33/ 7.31/ 13.16 | 5.64/ 6.31/ 9.02 |
| RANTES | 129.86/ 205.20/ 276.69 | 16415.91/ 20286.92/ 47153.37 | 5186.65/ 8990.94/ 16209.72 | 9748.80/ 14901.35/ 18717.77 | 1500.23/ 7438.98/ 11664.25 | 11357.38/ 13929.45/ 16988.22 |

Note: After the plasma sample was diluted, the concentrations of TNF-α, IFN-β and IL-6 in control were lower than the detection limit. The Dex group was not set for IL-6 and TNF-α.

Table S7. Relative transcript level of the mRNA of TNF-α, IL-1β, IL-6, IL-12B, MCP-1, and TGF-β in mesenteric lymph nodes of C57BL/6 mice after oral administration of BA, CBA-SME, and BAPC-SME following intraperitoneal injection of lipopolysaccharide for 1.5 h. The data are presented as the mean ± standard error of the mean (n=3).

| Group | Control | Model | Dex | BA | BA-SME | BAPC-SME |
| --- | --- | --- | --- | --- | --- | --- |
| TNF-α | 1.00±0.08 | 1.17±0.13 | 0.57±0.16 | 0.92±0.22 | 1.26±0.11 | 0.52±0.19 |
| TGF-β | 1.00±0.04 | 0.15±0.02 | 0.30±0.05 | 0.23±0.04 | 0.35±0.04 | 0.36±0.02 |
| MCP-1 | 1.00±0.13 | 29.99±2.08 | 32.04±5.44 | 45.32±16.02 | 43.45±11.91 | 10.57±4.15 |
| IL-6 | 1.00±0.26 | 198.39±15.64 | 34.40±11.40 | 292.95±35.34 | 123.55±6.72 | 9.32±4.95 |
| IL-1β | 1.00±0.20 | 28.53±0.69 | 6.85±0.27 | 32.67±14.43 | 20.62±1.37 | 6.79±4.70 |
| IL-12B | 1.00±0.14 | 5.84±0.90 | 1.64±0.30 | 5.72±1.57 | 3.77±0.84 | 0.97±0.13 |

Table S8. Relative mRNA level of TNF-α, MCP-1, IL-1β, IL-6, IL-10 and IFN-γ in the lung. The data are presented as the mean ± standard error of the mean (n = 4).

| Group | Control | Model | Dex | BA | BA-SME | BAPC-SME |
| --- | --- | --- | --- | --- | --- | --- |
| IL-1β | 1.02±0.12 | 7.65±1.54 | 2.92±0.13 | 8.76±2.38 | 5.27±1.75 | 4.39±1.81 |
| IL-6 | 1.01±0.05 | 22.05±6.14 | 4.42±0.65 | 19.08±2.60 | 14.25±12.05 | 3.71±2.14 |
| IL-10 | 1.01±0.07 | 10.07±0.23 | 9.83±0.21 | 6.59±0.20 | 4.63±1.21 | 2.65±0.56 |
| TNF-α | 1.02±0.08 | 1.82±0.21 | 1.62±0.08 | 1.21±0.04 | 0.96±0. 0.21 | 0.94±0.12 |
| MCP-1 | 1.10±0.25 | 49.27±10.80 | 7.51±0.54 | 27.04±2.31 | 22.19±7.15 | 8.58±2.00 |
| IFN-γ | 1.02±0.11 | 0.71±0.16 | 0.06±0.01 | 0.46±0.05 | 0.41±0.09 | 0.37±0.02 |

**Reference**

[1] EVERS D L, CHAO C F, WANG X, et al. Human cytomegalovirus-inhibitory flavonoids: Studies on antiviral activity and mechanism of action [J]. Antiviral Research, 2006, 68(3): 124-34.

[2] LYU S Y, RHIM J Y, PARK W B. Antiherpetic activities of flavonoids against herpes simplex virus type 1 (HSV-1) and type 2 (HSV-2) in vitro [J]. Arch Pharmacal Res, 2005, 28(11): 1293-301.

[3] FLEISCHMANN, CAROLIN, REINHART, et al. Assessment of Global Incidence and Mortality of Hospital-treated Sepsis [J]. American Journal of Respiratory & Critical Care Medicine, 2016, 193: 259-72.

[4] KUMAR A, ROBERTS D, WOOD K E, et al. Duration of hypotension before initiation of effective antimicrobial therapy is the critical determinant of survival in human septic shock [J]. Critical Care Medicine, 2006, 34(6): 1589-96.

1. ^^ Corresponding author: Professor Yuling Liu

   State Key Laboratory of Bioactive Substance and Function of Natural Medicines, Institute of Materia Medica, Chinese Academy of Medical Sciences & Peking Union Medical College, 1 Xiannongtan Street, Beijing 100050, People’s Republic of China

   Telephone: +86 108 928 5188

   Fax: +86 108 928 5190

   Email: ylliu@imm.ac.cn [↑](#footnote-ref-2)
